# The S180R Human Germline Variant of DNA Polymerase β Is a Low Fidelity Enzyme with Reduced Flexibility of the Fingers Domain

**DOI:** 10.1021/acs.biochem.5c00628

**Published:** 2026-01-12

**Authors:** Danielle L. Sawyer, Brian E. Eckenroth, Cristian Chavira, Khadijeh Alnajjar, John P. Hanley, Julie A. Dragon, Sylvie Doublié, Joann B. Sweasy

**Affiliations:** † Department of Cell and Molecular Medicine, 12216 University of Arizona, 1515 N Campbell Avenue, Tucson, Arizona 85724, United States; ‡ Department of Microbiology and Molecular Genetics, 2092 University of Vermont, Stafford Hall, 95 Carrigan Drive, Burlington, Vermont 05405, United States; § Eppley Institute for Research in Cancer and Allied Diseases, Fred & Pamela Buffett Cancer Center, 12284 University of Nebraska Medical Center, Omaha, Nebraska 68198, United States

## Abstract

DNA polymerase β (Pol β) is an important polymerase that functions in DNA repair within the Base Excision Repair and Non-Homologous End-Joining pathways. It is estimated to function in the repair of up to 50,000 DNA lesions per cell per day, within the base excision repair pathway (BER). Given the significant role Pol β plays in repairing DNA, genetic variants of Pol β have the potential to perturb repair, resulting in mutation accumulation which can potentiate cancer formation. Here we identify an unstudied human germline variant of Pol β, the S180R variant (rs1585898410), which introduces a significant amino acid alteration within the dNTP binding pocket of the enzyme. We demonstrate that S180R is a low fidelity variant of Pol β due to its loss of the ability to discriminate correct nucleotides from incorrect nucleotides. We also show that this variant exhibits a much slower rate of nucleotide incorporation, which could further disrupt repair capacity *in vivo*. Structural data reveal that this variant not only has structural changes that may disrupt dNTP binding but also a loss of primer terminus positioning and dynamic flexibility of the fingers domain in the binary state, which likely are driving the low fidelity of S180R Pol β. This study highlights the importance of binary positioning and nucleotide coordinating residues for maintaining nucleotide selectivity, polymerase function, and fidelity. It also emphasizes the importance of further study of this human germline Pol β variant *in vivo*.

## Graphical abstract

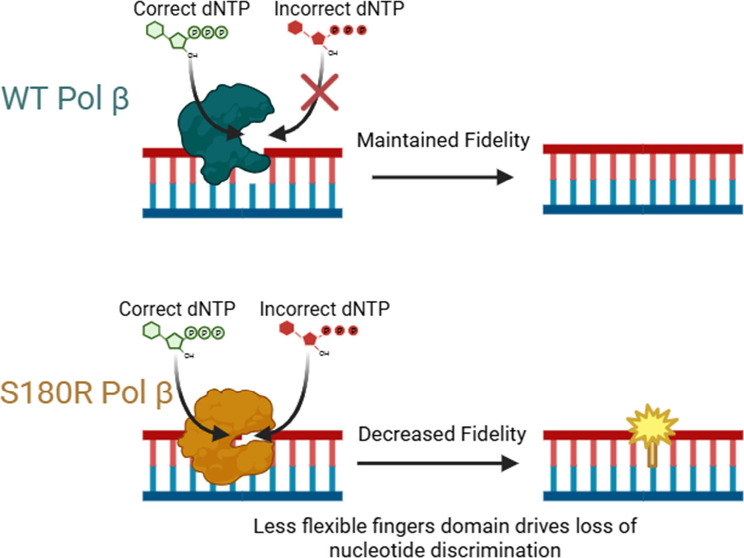

## Introduction

DNA polymerase β (Pol β) is a small mammalian polymerase with a critical role in DNA repair in the base-excision repair pathway, where Pol β incorporates a nucleotide into a single nucleotide gapped DNA substrate.^1–3^ Pol β has a massive repair responsibility, as it functions in the base excision repair pathway that is accountable for the repair of up to 50,000 DNA base lesions per cell per day.^4^ As Pol β lacks a proofreading exonuclease domain,^5,6^ it is critical that it selects the correct nucleotide for incorporation into DNA to avoid accumulation of mutations. Somatic variants of Pol β are found in tumor samples, suggesting that variant forms of Pol β may be playing a role in driving spontaneous cancer formation.^7,8^ However, only recently has research begun to explore the effect of germline variants of Pol β, which may represent a heritable risk for cancer or other human diseases.

Pol β has two domains, a lyase domain with 5′dRP lyase activity as well as a polymerase domain responsible for nucleotidyl transferase activity.^5,6,9^ The polymerase domain is divided into three subdomains: the thumb, palm, and fingers, based upon the nomenclature originally proposed by Steitz.^10,11^ The fingers subdomain closes following correct dNTP binding to form the complete active site.^5,6,9,10,12–15^ Pol β is thought to have at least eight known steps in its catalytic pathway.^16^ After DNA and dNTP substrate binding, the fingers subdomain closes (fingers closing step) and another precatalytic noncovalent step occurs,^13^ which is thought to include critical active site residue adjustments^17^ but is not well understood. Following these conformational changes, nucleotidyl transfer takes place, requiring at least two magnesium ions cofactors.^18–20^ After phosphodiester bond formation, the fingers reopen and release the DNA and pyrophosphate product.^13,21^ Importantly, residue mutations that disrupt some of these steps, such as the noncovalent and fingers closing step, highlight the importance of these dynamic movements for maintaining Pol β function and fidelity.^22–24^

We have identified the S180R variant of Pol β (rs1585898410), which is a rare human germline mutation that is present within cancer patient databases (refer to the Materials and Methods) and has recently been identified in the Korean Genome Project at a reported frequency of 0.38%. We found this variant of interest for further characterization, as the S180 residue is within the palm subdomain of Pol β and is known to form a hydrogen bond with the γ-phosphate of the incoming dNTP following the fingers closing step.^5,6,25^ Importantly, adding a positively charged arginine within the dNTP binding pocket of Pol β could significantly disrupt the Pol β function. Residues S180, R183, and R149 coordinate the β- and γ-phosphate groups of the incoming dNTP **(** Figure 1). Molecular dynamics simulations suggest that residues S180 and R183 interact with the incoming nucleotide and pull it away from the primer end in the absence of the catalytic magnesium, and are thought to facilitate Pol β fingers opening.^26^ Kraynov et al.’s study of the S180A mutation shows a decreased rate of polymerization and dNTP binding affinity and suggests the importance of the S180 hydroxyl group in stabilizing the dNTP in the active site.^27^

**Figure 1:**
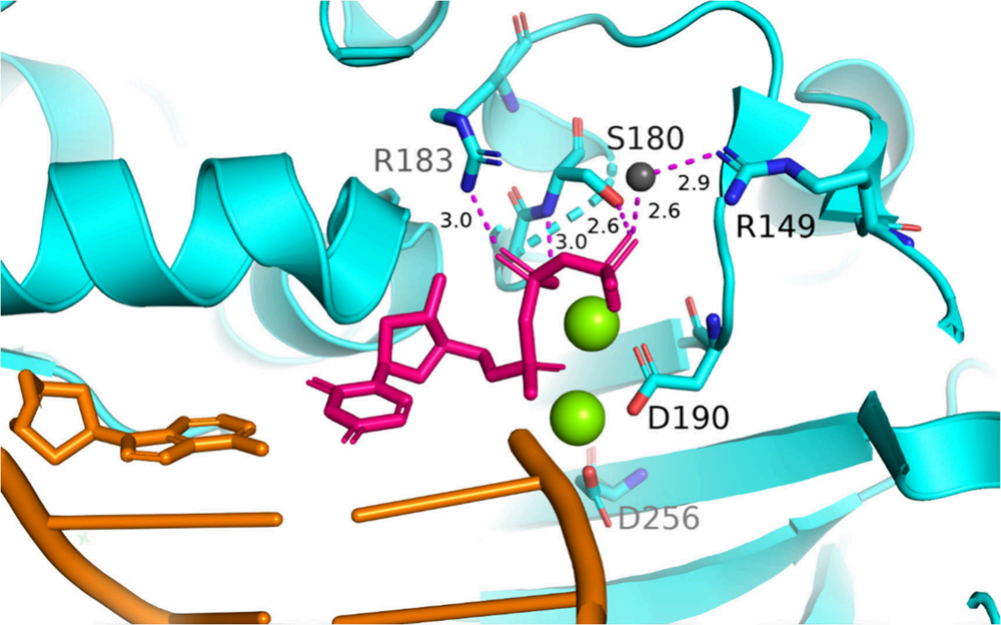
Crystal structure of WT ternary Pol β (PDB: 4KLD), with Pol β in cyan, DNA template in orange, and incoming nucleotide in pink. Green spheres represent divalent metal ions, coordinated by D190, D192 (not shown), and D256, which are required for the reaction to proceed. Gray sphere represents a water molecule that coordinates with the gamma phosphate of the incoming nucleotide. Nucleotide coordinating residues are shown (R149, S180, and R183), with dashed lines representing distances of interactions between the residues and β- and γ-phosphate groups.

The S180R human variant of Pol β represents a variant with a dramatic residue substitution, and studies herein illustrate the effect of this human germline mutation on the function of Pol β. We investigated biochemically and structurally the function of S180R in comparison with the WT enzyme for all four templating base options. In this study, we observed that the S180R variant of Pol β exhibits a low fidelity phenotype as a result of decreased discrimination for the correct nucleotides. We also demonstrate that this variant has a slower rate of nucleotide incorporation and a slower rate of turnover during steady-state nucleotide incorporation. Additionally, crystal structure data show that the S180R mutation disrupts residues and water molecules, which help to coordinate the incoming phosphate group. The structural data also demonstrate that this low-fidelity variant has decreased dynamic movements of the fingers domain and altered primer positioning during nucleotide selection. Together, our data suggest that S180R results in a loss of nucleotide discrimination and that the binary state and dynamics are crucial for maintaining fidelity. These results are consistent with the possibility that the S180R germline variant has the potential to disrupt DNA repair *in vivo*.

## Materials and Methods

### Bioinformatics for Pol β Variants

Germline variants of Pol β were selected using the Genomic Data Commons (GDC) Application Programming Interface (API) and by selecting subjects within The Cancer Genome Atlas (TCGA) with at least one normal and one cancer sample as described.^28^ Within the TCGA database the S180R germline variant of Pol β exists at a low frequency (0.79% of overall samples with 4.86% in kidney renal clear cell carcinoma, 2.71% in glioblastoma multiforme, 1.98% in lung squamous cell carcinoma,1.91% in breast invasive carcinoma, 0.70% in ovarian serous cystadenocarcinoma, 0.68% in stomach adenocarcinoma, 0.53% in liver hepatocellular carcinoma, 0.43% colon adenocarcinoma, 0.40% in prostate adenocarcinoma, 0.34% in lung adenocarcinoma, 0.34% in kidney renal papillary cell carcinoma, 0.20% in thyroid carcinoma, and 0.19% in head and neck squamous cell carcinoma). Otherwise, the frequency was 0% in other cancer types. The S180R germline variant has also been identified as a single nucleotide polymorphism (SNP) rs1585898410 is listed under NCBI as having a frequency of 0.0038 (*n* = 7/1832 of the Korea1K genomes project).

### Protein Purification

DNA polymerase β’s UniProt ID is P06746. The Q5 Site directed mutagenesis kit (NEB E0554S) was used to introduce the S180R residue change into the pET28a-Human WT Pol β-tagless expression vector as described.^23,24^ Plasmid Pol β cDNA sequence was verified by DNA sequencing (University of Arizona Genetics Core). Plasmids expressing WT or S180R Pol β were transformed into Rosetta (DE3) cells for recombinant protein expression and selected with Kanamycin + Chloramphenicol. Proteins were expressed and purified as previously described.^29^ Protein was stored in 15% glycerol at −80 °C.

### DNA for Kinetic Assays

DNA oligomers were ordered from Integrated DNA Technologies (IDT) and purified using gel purification. The oligo upstream of the single nucleotide gap was radiolabeled with 5′P-32 using T4 PNK (NEB entry M0201) for DNA product visualization. The downstream oligo contained a 5′-phosphate modification, which is crucial for Pol β activity as described.^30^ The three DNA oligos (upstream, downstream, and templating oligo) were combined and annealed using a temperature ramp with a thermocycler to form a DNA duplex with a single nucleotide gap (sngDNA). Annealed DNA was confirmed by running a 12% native gel and using recessed DNA as a negative control. DNA sequences used are shown below in Figure 2. C-template was the only sequence modified to avoid strand slippage during polymerase nucleotide incorporation, with the downstream base changed to G.

**Figure 2:**
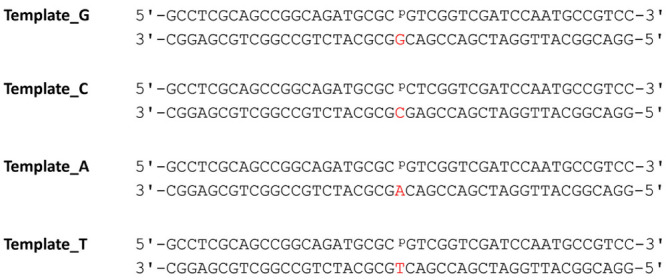
Single nucleotide gapped DNA sequences used for kinetic experiments, with templating bases in red.

### Circular Dichroism

Purified WT Pol β and S180R Pol β proteins were resuspended to a final concentration of 3 μM in a phosphate buffering solution (10 mM dibasic potassium phosphate, pH 8.0). An Olis DSM-20 CD instrument was used to measure the absorbance of circular polarized light at 208 nm (alpha helical^24^), with temperature increasing from 20–50 °C at 1 °C increment steps. The degree of ellipticity at each temperature point was averaged from the value before and after equilibrating for 1 min. Melting temperature (*T*_m_) was estimated by fitting the points to a sigmoidal equation using GraphPad Prism, as described.^24^

### Kinetic Assays

For the burst assays, the Kintek Rapid Quench Flow (RQF) instrument was used to mix reactants and rapidly quench each reaction with EDTA at specified time points as described.^13,31^ To ensure that biphasic burst conditions were present, the ratio of protein to DNA was held constant at 1:3, respectively. The RQF temperature was regulated by a recirculating water pump set to 25 °C. The Kintek high salt (KHS) reaction buffer was used (50 mM Tris Cl, pH 8, 100 mM NaCl, 2 mM DTT, 10% glycerol). For burst conditions: Protein/DNA preformed complex was mixed with dCTP (final concentration at 100 nM Pol β, 300 nM G-template sngDNA and 100 μM dCTP, 10 μM MgCl_2_, in KHS). The reaction was quenched with 0.5 M EDTA and the product was resolved on a 20% sequencing gel (19:1 acrylamide:bisacrylamide and 7.5 M Urea, SequaGel Urea Gel system: EC-833). Gels were exposed to phosphor screens, imaged using a Typhoon phosphor imager (Amersham-Cytiva) and quantified using ImageQuant to calculate product formation. The data were fitted to the biphasic burst equation (Equation 1) as described^31–33^ using GraphPad Prism.

$$\left[product\right]={\left[E\right]}_{app}\left(\frac{k_{obs}}{{\left(k_{obs}+k_{ss}\right)}^{2}}\left(1-e^{-{\left(k_{obs}+k_{ss}\right)}^{t}}\right)+\frac{\left(k_{obs}k_{ss}\right)}{\left(k_{obs}+k_{ss}\right)}t\right)$$ 1

For single turnover kinetics (STN), the ideal ratio of protein:DNA was confirmed for S180R and WT by empirical determination under single turnover conditions as described.^30,34^ This ideal ratio was confirmed to be the standard 15:1 ratio for both WT and S180R. Using the Kintek RQF instrument at 25 °C, reactant mixes containing Pol β/DNA and variable dNTP concentrations were mixed at a 1:1 ratio (final concentration: 750 nM protein, 50 nM sngDNA, 50 mM MgCl_2_, and dNTP concentrations varied from 0 to 1200 μM, in KHS reaction buffer). Reactions were performed and then quenched with 0.5 M EDTA at specific time points, and the reaction solution was collected, all as previously described.^35–37^ The reactions were resolved on a sequencing gel as described above, and product formation versus time was plotted as a single exponential equation in Prism to determine a *k*_obs_ rate for each concentration of dNTP. *k*_obs_ rates for each concentration were then fit to a hyperbolic equation to determine *K*_d(dNTP)_ and *k*_pol_ values, as described.^32^

For incorrect STN assays, reactant mixes of Protein/DNA and dNTP (dNTP concentration ranged 0–5000 μM) as described above were mixed at 25 °C. Reactions were manually quenched with 0.5 M EDTA at the specified time points, all as described previously.^35–37^ The reactions were resolved on a sequencing gel and plotted as described for the correct STN kinetics.

### Discrimination Calculations

*K*_d(dNTP)_ and *k*_pol_ values were calculated from Prism as described above and used to calculate the efficiency (Equation 2) for both correct and incorrect pairings. Discrimination at the level of *K*_d(dNTP)_ and *k*_pol_, and fidelity were then calculated (eqs 3, 4, and 5, respectively). Using fidelity for both WT and the S180R variant, with X-fold value, a ratio of WT fidelity to variant fidelity was determined (eq 6). All calculations are described previously in the literature.^24,32^

2 $$Efficiency=\frac{k_{pol}}{K_{d\left(\mathrm{dNTP}\right)}}$$

3 $$Discrimination\;\left(K_{d\left(\mathrm{dNTP}\right)}\right)=\frac{K_{d\left(\mathrm{incorrect}\right)}}{K_{d\left(\mathrm{correct}\right)}}$$

4 $$Discrimination\;\left(k_{pol}\right)=\frac{k_{pol\left(correct\right)}}{k_{pol\left(incorrect\right)}}$$

5 $$Fidelity=\frac{{Efficiency}_{correct}+{Efficiency}_{incorrect}}{{Efficiency}_{incorrect}}$$

6 $$X\text{‐}fold=\frac{{Fidelity}_{WT}}{{Fidelity}_{Variant}}$$

### Electrophoretic Mobility Shift Assay (EMSA)

WT or S180R Pol β was titrated (0.25–2000 nM) in the presence of 0.1 nM G-template sngDNA. The reaction was conducted in binding buffer (10 mM Tris (pH 7.6) and 8 mM MgCl_2_, 100 mM NaCl, 10% glycerol, and 0.1% NP-40 (IGEPAL)). The reaction mixtures were resolved on a 10% native acrylamide gel and Hoefer gel system at 4 °C as previously described.^30,38^ The gels were dried and exposed to phosphor screens. Images were captured using a Typhoon phosphor imager and quantified using ImageQuant. Percent complex formation was plotted as a function of Pol β concentration, and the data were fit to a specific binding with hill slope equation using GraphPad Prism as described.^30^ Mean *K*_d(DNA)_ values and SEM were used in an unpaired two-sided *t* test to determine whether the mean values were significantly different from one another. EMSA studies for Pol β are unable to measure a true *K*_d_ value, as they do not consider protein activity and do not calculate a true *K*_d(DNA)_. They were used as a straightforward way to estimate protein–DNA interactions and compare variant to WT protein interactions to calculate an apparent *K*_d_ value.

### Sample Preparation and Crystallization

Protein expression was performed in *E. coli* using a pET28 vector with a stop codon inserted to omit the His-tag. Purification was conducted using a three-column protocol of DEAE Sepharose, heparin and SP Sepharose, as previously described^39^ with the purified sample concentrated to 25–30 mg/ml and stored at −80 °C. The DNA sequence context used in crystallization was as follows: 5′-CCGAC­(X)­GCGCATCAGC-3′ template where (X) was dA, dG, dT, or dC, 5′-GCTGATGCGC-3′ primer, and 5′-Phos-GTCGG-3′ downstream. The oligonucleotides used in crystallization were synthesized by Integrated DNA Technologies. (Coralville, IA), PAGE purified, and mixed in a 1:1:1.2 ratio. The oligonucleotides were annealed by heating to 90 °C for 10 min, allowed to cool to room temperature, and then incubated on ice for 10 min prior to use. The binary complexes bound to single nucleotide gapped DNA substrate were prepared by mixing to 250 μM Pol β and 300 μM DNA. Crystallization wells contained 13–16% PEG 3350, 150–300 mM sodium acetate with HEPES pH 7.5, and 1 mM TCEP and were incubated at 18 °C upon mixing equal volume binary complex with reservoir solution. Cryoprotection was achieved by bringing the final PEG 3350 concentration to 19% with the addition of 15% ethylene glycol in three steps then bringing the sodium acetate to 150 mM with 50 mM magnesium chloride and 50 mM magnesium acetate prior to flash cooling in liquid nitrogen.^40^ The ternary complexes were prepared by inclusion of 1 mM dNTP dictated by the templating base via soaking of binary form crystals. The nonhydrolyzable dNTP analogues dUmpNpp, dAmpCpp, dCmpCpp, and dGmpCpp were purchased from Jena Biosciences (Jena, Germany). Binary and ternary complexes all use the same reagent to reduce the potential differences in ion content. Data index and integration using Dials^41^ or Mosflm,^42^ scaling and merging using Aimless^43^ within CCP4 ^44^ for synchrotron data sets or SAINT and SADABS within Proteum3 (Bruker-AXS) for in-house crystallographic data sets.

### Structure Determination

Data were checked for isomorphism with WT binary and ternary complexes using PDB ID 3ISB^45^ or PDB ID 2FMS,^40^ respectively and used for isomorphous difference Fourier methods^46,47^ The degree of isomorphism between S180R data sets and 3ISB (binary complex) and 2FMS (ternary complex) reference data sets was found to be unsatisfactory for isomorphous difference Fourier methods,^46^ which led to the data collection of both binary and ternary WT Pol β, with all four templating bases (eight data sets total), to be used as reference data sets. All solvent molecules, incoming nucleotide, and templating bases were removed from the starting model prior to map calculation, building, and refinement. All model building was performed using Coot^48^ and refinement performed using Phenix.^49^ Refinement schemes included defined domain-based TLS refinement. Structural figures were prepared using PyMOL (Schrödinger Inc.) and data processing and model refinement are summarized in Tables 1, 2, and 3. Isomorphous difference Fourier maps^50^ were calculated using Phenix using Fobs and equivalent resolution range of 20–2.1 Å with correlation coefficients extracted. B-factor analysis was performed using the B-Average^51^ utility within CCP4. Correlation coefficients between two crystallographic data sets were calculated within Phenix using eq 7,

$$CC=\frac{\underset{hkl}{\sum }\left(\right|F_{1}\left(hkl\right)|-|\overset{¯}{F_{1}}|)\left(|F_{2}\left(hkl\right)|-|\overset{¯}{F_{2}}|\right)}{\sqrt{{{\underset{hkl}{\sum }\left(\right|F_{1}\left(hkl\right)|-|\overset{¯}{F_{1}}|)}^{2}\underset{hkl}{\sum }\left(\right|F_{2}\left(hkl\right)|-|\overset{¯}{F_{2}}|)}^{2}}}$$ 7

where |*F*_1_(*hkl*)| and |*F*_2_(*hkl*)| are the structure factor amplitudes for the two crystal structures for reflection *hkl*, and |*F̅*_1_| and |*F̅*_2_| are the mean amplitudes for each data set.

**Table 1:** Single Turnover Data for S180R vs WT^a^

sequence	protein	*k*_pol_ (s^–1^)	*K*_d(dNTP)_ (μM)	discrimination *k*_pol_ ^a^	discrimination *K*_d_ ^b^	efficiency (μM^–1^ s^–1^)^c^	fidelity^d^	X-fold^e^
**C:dGTP**	**WT**	2.3 ± 0.33	1.0 ± 0.14	-	-	2.45E+00	-	**-**
	**S180R**	0.82 ± 0.17	58 ± 3.1	-	-	1.40E–02	-	
**C:dCTP**	**WT**	0.0056 ± 0.0008	530 ± 38	420	555	1.05E–05	2.33E+05	**8.40**
	**S180R**	0.00043 ± 0.00003	860 ± 17	1880	15	5.05E–07	2.78E+04	
**C:dATP**	**WT**	0.031 ± 0.004	92 ± 1.4	75	96	3.38E–04	7.26E+03	**9.43**
	**S180R**	0.0020 ± 0.0001	110 ± 10	407	2	1.82E–05	7.70E+02	
**C:dTTP**	**WT**	0.019 ± 0.003	376 ± 40	125	393	4.98E–05	4.92E+04	**1.95**
	**S180R**	0.00017 ± 0.000001	308 ± 26	4762	5	5.57E–07	2.52E+04	
**G:dCTP**	**WT**	3.9 ± 0.14	2.1 ± 0.06	-	-	1.84E+00	-	**-**
	**S180R**	0.92 ± 0.16	96 ± 13	-	-	9.60E–03	-	
**G:dATP**	**WT**	0.0029 ± 0.0004	83 ± 10	1335	39	3.51E–05	5.24E+04	**19.1**
	**S180R**	0.00068 ± 0.00008	195 ± 34	1346	2	3.51E–06	2.74E+03	
**G:dGTP**	**WT**	0.0013 ± 0.0005	294 ± 37	3070	139	4.33E–06	4.25E+05	**x**
	**S180R**	x	x	x	x	x	x	
**G:dTTP**	**WT**	0.028 ± 0.003	420 ± 64	141	198	6.59E–05	2.80E+04	**7.14**
	**S180R**	0.00059 ± 0.00018	240 ± 46	1564	3	2.45E–06	3.91E+03	
**T:dATP**	**WT**	3.0 ± 0.30	0.31 ± 0.001	-	-	9.68E+00	-	**-**
	**S180R**	1.1 ± 0.15	91 ± 8.4	-	-	1.21E–02	-	
**T:dCTP**	**WT**	0.023 ± 0.003	281 ± 35	133	903	8.06E–05	1.20E+05	**62.0**
	**S180R**	0.0011 ± 0.00011	170 ± 30	1031	2	6.26E–06	1.94E+03	
**T:dGTP**	**WT**	0.069 ± 0.011	206 ± 29	43	662	3.37E–04	2.87E+04	**39.8**
	**S180R**	0.0015 ± 0.00023	91 ± 9	719	1	1.68E–05	7.21E+02	
**T:dTTP**	**WT**	0.0047 ± 0.0011	378 ± 2.4	636	1211	1.26E–05	7.71E+05	**68.7**
	**S180R**	0.00022 ± 0.000023	199 ± 16	5100	2	1.08E–06	1.12E+04	
**A:dTTP**	**WT**	6.7 ± 1.3	7.4 ± 1.3	-	-	8.97E–01	-	**-**
	**S180R**	1.1 ± 0.14	405 ± 19	-	-	2.72E–03	-	
**A:dCTP**	**WT**	0.016 ± 0.00088	314 ± 20	427	42	4.97E–05	1.81E+04	**31.8**
	**S180R**	0.0011 ± 0.000065	233 ± 16	989	0.6	4.79E–06	5.68E+02	
**A:dGTP**	**WT**	0.00055 ± 0.000047	653 ± 109	12,081	88	8.45E–07	1.06E+06	**x**
	**S180R**	x	x	x	x	x	x	
**A:dATP**	**WT**	0.0021 ± 0.00005	54 ± 8	3140	7	3.94E–05	2.28E+04	**27.2**
	**S180R**	0.00024 ± 0.00007	74 ± 12	4592	0.2	3.25E–06	8.36E+02	

a Column 1 shows the templating base of the sngDNA and the incoming dNTP. Column 3 represents rate of nucleotide incorporation (*k*_pol_), and column 4 shows apparent nucleotide dissociation constant (*K*_d_). Discrimination at the level of *k*_pol_ and *K*_d_, as well as efficiency, fidelity, and X-fold values for each pairing, are calculated based on indicated equations. *K*_d_ and *k*_pol_ values are reported as the mean ± standard error for two or more replicates.

b Discrimination (*k*_pol_) = *k*_pol_(correct)/*k*_pol_(incorrect).

c Discrimination (*K*_d_) = *K*_d(dNTP)_(incorrect)/*K*_d(dNTP)_(correct).

d Efficiency = (*k*_pol_/*K*_d(dNTP)_).

e Fidelity = (Correct Efficiency + Incorrect Efficiency)/(Incorrect Efficiency).

f X-fold = WT Fidelity/S180R Fidelity.

**Table 2:** Crystallographic Data Collection and Structure Refinement Statistics for the Pol β S180R

	binary				ternary			
	dA	dG	dT	dC	dA:dUmpNpp	dG:dCmpCpp	dT:dAmpCpp	dC:dGmpCpp
**PDB ID**	9Y1D	9Y1E	9Y1F	9Y1G	9Y1H	9Y1I	9Y1J	9Y1K
**Diffraction**	Diamond I24	Diamond I24	Diamond I24	Diamond I24	Bruker	Bruker	Diamond I24	Diamond I24
**Data processing**	Mosflm	Dials	Dials	Dials	Proteum	Proteum	Dials	Mosflm
**Cell** ***a*, *b*, *c* (Å)**	54.3, 79.3, 54.9	54.5, 79.6, 55.0	54.2, 79.3, 54.6	54.4, 79.8, 55.1	50.4, 79.2, 55.5	50.8, 79.9, 55.4	50.8, 79.2, 55.5	50.6, 80.1, 55.5
**β** **(** ^**o**^ **)**	105.7	105.6	105.1	105.3	107.3	107.5	107.6	107.5
**Resolution (Å) (high resolution)** ^a^	45–1.70 (1.73–1.70)	44–1.60 (1.63–1.60)	45–1.80 (1.84–1.80)	44–1.60 (1.63–1.60)	27–1.96 (2.03–1.96)	25–2.15 (2.22–2.15)	44–1.55 (1.58–1.55)	48–1.70 (1.73–1.70)
**Wilson B**	19.2	25.1	23.2	24.4	21.5	21.1	14.3	22.3
**Reflections**	49130	59235	41360	59677	30406	28561	60713	46469
**Completeness (%)**	99.5 (98.7)	99.6 (92.0)	99.8 (99.6)	99.5 (92.0)	98.9 (90.2)	98.9 (89.4)	99.8 (96.1)	99.9 (99.9)
**Multiplicity**	3.3 (3.1)	6.4 (5.1)	6.4 (6.6)	6.2 (5.1)	4.7 (2.1)	3.9 (2.7)	6.3 (4.2)	6.1 (5.9)
***I*/sig*I***	7.4 (2.2)	7.2 (0.6)	8.9 (1.8)	7.4 (0.7)	18.7 (2.1)	17.8 (3.6)	15.6 (2.4)	6.2 (1.0)
***R***_**meas**_ **%**	9.9 (46.3)	11.1 (99.7)	14.2 (128)	11.1 (76.8)	6.2 (53.6)	6.8 (36.6)	7.4 (56.0)	17.2 (211)
***R***_**pim**_ **%**	5.2 (25.3)	4.4 (44.0)	5.8 (54.5)	4.5 (34.0)	2.7 (32.4)	3.4 (20.4)	2.9 (26.5)	7.6 (95.0)
**CC** ^**1/2**^	0.992 (0.784)	0.996 (0.581)	0.988 (0.430)	0.997 (0.691)	0.998 (0.736)	0.998 (0.862)	0.999 (0.739)	0.987 (0.222)
**model *R***_**work**_ **/*R***_**free**_ **%**	15.9/19.5	18.1/20.6	16.9/20.2	17.7/20.9	15.3/20.2	15.6/21.3	14.9/18.3	17.3/21.7
**RMS bonds (Å)**	0.011	0.007	0.007	0.007	0.006	0.004	0.012	0.007
**RMS angles (** ^**o**^ **)**	1.224	0.903	0.914	0.893	0.843	0.669	1.306	0.920
**Ramachandran (%)**								
Favored	97.8	97.8	98.1	98.1	99.1	99.4	98.5	98.5
Outlier	0	97.8	98.1	98.1	99.1	99.4	98.5	98.5
**Mean B (Å** ^**2**^ **)**	28.4	33.4	29.9	33.4	27.6	26.2	20.9	31.0
**Correlation coefficient**	0.903	0.897	0.891	0.888	0.917	0.918	0.920	0.889
**Atoms protein**	2590	2590	2577	2602	2610	2613	2519	2617
**DNA**	631	632	630	629	631	631	630	629
**Ligand/ion**	3	4	4	3	33	33	34	35
**Solvent**	580	493	494	567	416	384	674	465
**Coordinate error (Å)**	0.20	0.27	0.21	0.24	0.20	0.23	0.15	0.23
**Phase error (** ^**o**^ **)**	19.0	21.9	19.4	21.9	20.0	21.4	17.1	23.3
***R***_**Cross**_ **(%) WT** ^b^	21.7	23.9	17.7	25.4	13.5	12.7	12.2	16.6

a Numbers in parentheses denote high resolution bin.

b The overall data isomorphous R-factor (*R*_iso_) as calculated on *F*_obs_ using Scaleit to the equivalent WT complex from Table 3.

**Table 3:** Crystallographic Data Collection and Structure Refinement Statistics for Pol β WT

	binary				ternary			
	dA	dG	dT	dC	dA:dUmpNpp	dG:dCmpCpp	dT:dAmpCpp	dC:dGmpCpp
**PDB ID**	9Y15	9Y16	9Y17	9Y18	9Y19	9Y1C	9Y1A	9Y1B
**Diffraction**	Bruker	Bruker	Bruker	Bruker	Bruker	Bruker	Bruker	Bruker
**Data processing**	Proteum	Proteum	Proteum	Proteum	Proteum	Proteum	Proteum	Proteum
**Cell *a*, *b*, *c* (Å)**	54.3, 78.6, 54.8	54.0, 79.0, 54.5	54.0, 79.0, 54.5	54.1, 78.5, 54.7	50.7, 79.3, 55.5	50.7, 79.7, 55.5	50.6, 79.0, 55.6	50.5, 79.3, 55.3
**β** **(** ^**o**^ **)**	104.6	104.9	104.4	104.4	107.2	107.4	107.4	107.3
**Resolution (Å) (high resolution)** ^a^	25–1.85 (1.92–1.85)	25–1.80 (1.86–1.80)	25–1.85 (1.92–1.85)	25–1.80 (1.86–1.80)	25–1.90 (1.97–1.90)	25–1.90 (1.97–1.90)	25–1.93 (2.00–1.93)	25–1.90 (1.97–1.90)
**Wilson B**	16.3	18.1	16.5	14.8	17.4	17.3	17.8	20.2R
**Reflections**	37983	40929	37956	41064	32756	33091	31357	32674
**Completeness (%)**	99.7 (99.2)	99.9 (99.4)	100 (99.9)	100 (99.7)	99.0 (96.9)	99.6 (99.6)	99.7 (98.4)	99.6 (99.2)
**Multiplicity**	5.3 (3.5)	8.4 (6.3)	5.5 (4.4)	6.9 (4.9)	3.4 (2.1)	3.9 (2.8)	3.7 (2.7)	5.1 (3.3)
***I*/sig*I***	18.1 (3.4)	22.6 (4.7)	19.2 (5.0)	22.6 (6.0)	14.2 (3.0)	12.1 (2.7)	13.6 (2.6)	9.7 (1.1)
***R***_**meas**_ **%**	6.0 (45.4)	5.9 (46.0)	6.0 (33.0)	5.7 (28.9)	6.1 (38.0)	8.0 (55.3)	8.7 (57.9)	10.2 (108.4)
***R***_**pim**_ **%**	2.3 (21.5)	1.9 (18.2)	2.4 (15.5)	2.0 (12.9)	2.9 (23.6)	3.7 (31.5)	4.4 (33.4)	4.7 (65.7)
**CC** ^**1/2**^	0.999 (0.866)	0.999 (0.916)	0.999 (0.933)	0.999 (0.946)	0.998 (0.846)	0.997 (0.786)	0.997 (0.720)	0.996 (0.539)
**Model *R***_**work**_ **/*R***_**free**_ **%**	16.8/21.2	16.9/20.9	16.6/20.8	16.3/20.5	14.8/19.7	15.4/19.8	15.5/20.1	18.6/22.5
**RMS bonds (Å)**	0.007	0.007	0.007	0.006	0.0113	0.006	0.007	0.005
**RMS angles (** ^**o**^ **)**	0.906	0.864	0.884	0.850	1.114	0.848	0.889	0.738
**Ramachandran (%)**								
Favored	98.4	98.1	98.1	98.1	98.8	99.7	98.5	99.1
Outlier	0	98.1	98.1	98.1	98.8	99.7	98.5	99.1
**Mean B (Å** ^**2**^ **)**	23.9	27.2	24.0	21.3	21.2	23.7	22.2	30.0
**Correlation coefficient**	0.917	0.912	0.915	0.922	0.926	0.925	0.926	0.901
**Atoms protein**	2567	2556	2526	2533	2614	2614	2612	2610
**DNA**	631	632	630	629	631	632	630	629
**Ligand/ion**	4	4	3	4	33	33	35	37
**Solvent**	582	556	571	635	505	571	502	415
**Coordinate error (Å)**	0.19	0.19	0.18	0.18	0.19	0.19	0.20	0.23
**Phase error (** ^**o**^ **)**	20.8	21.2	20.7	20.0	19.5	19.5	19.5	23.2
**PDB alternate**		3ISB			2FMS	5UGP		

a Numbers in parentheses denote high resolution bin.

## Results

### S180R Pol β Exhibits a Lower Melting Temperature than WT Pol β

To examine the *in vitro* stability of the S180R variant relative to WT, we employed circular dichroism to calculate the melting temperatures at 208 nm which is the maximum negative ellipticity of an alpha helical protein.^24,52^ The S180R residue mutation reduces the protein melting temperature from 37.9 °C for the WT to 34.1 °C, as shown in Figure 3. In order to study S180R protein kinetics without the influence of protein instability or unfolding, we performed all kinetic assays at 25 °C, where both the WT and S180R are completely folded.

**Figure 3:**
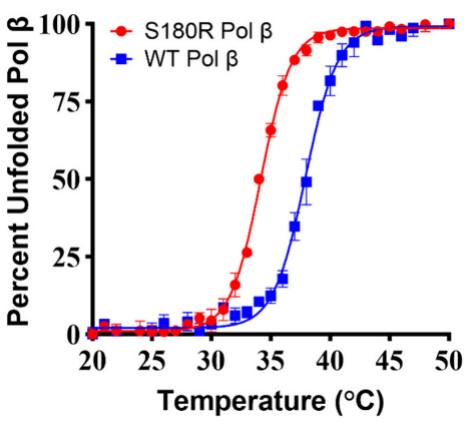
S180R Pol β has a lower melting temperature of 34.1 ± 0.05 °C compared to that of WT Pol β at 37.9 ± 0.1 °C. Percent unfolded protein calculated based on degree of ellipticity at 208 nm with increasing temperature. Plot of percent unfolding versus temperature was used to estimate melting temperature. Graph depicts mean values of percent unfolded ± standard error, for a minimum sample size of two per condition.

### S180R Binds DNA with a Similar Affinity as WT

Before performing kinetic studies to estimate rates of activity for the S180R variant relative to WT, we wanted to examine whether the S180R residue mutation impacts the affinity of Pol β for its DNA substrate. Therefore, we conducted an electromobility shift assay to estimate an apparent *K*_d(DNA)_ for S180R and WT (Figure 4), as described in the materials and methods. Both WT and S180R apparent *K*_d(DNA)_ values (WT = 28.8 ± 2.7 nM and S180R = 23.0 ± 2.3 nM) are not statistically different (*p = 0.23*) suggesting that S180R binds single nucleotide gapped DNA (sngDNA) with an affinity similar affinity as WT.

**Figure 4:**
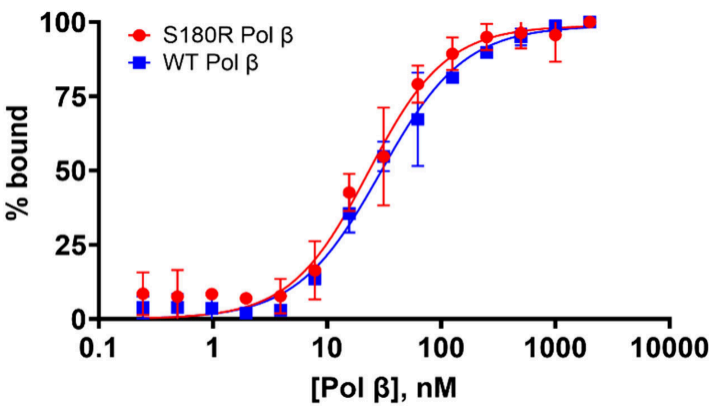
S180R Pol β has a similar affinity to single nucleotide gapped DNA as WT Pol β (*K*_d(DNA)_ = 23 ± 2.3 nM and *K*_d(DNA)_ = 28.8 ± 2.7 nM, respectively). Varied concentrations of S180R or WT Pol β (0.25–2000 nM) were mixed with 0.1 nM single nucleotide gapped DNA (G-template) and resolved on a native gel. Electromobility shift assay plot of percent bound (protein–DNA) complex versus Pol β concentration. Graph a plot of the mean values ± standard error of the mean for a minimum sample size of two per condition.

### S180R Exhibits Slow Biphasic Burst Kinetics

Under conditions of excess DNA, WT Pol β exhibits a biphasic burst where the first phase estimates the presteady state rate of turnover and is seen as rapid “burst” of activity (*k*_obs_) and the rate limiting step is phosphodiester bond formation.^53^ The second phase of the biphasic burst relates to steady-state dynamics (*k*_ss_) where DNA or pyrophosphate product release becomes rate-limiting.^53^ Both the WT and S180R exhibit a biphasic burst at 25 °C (Figure 5). S180R exhibits a 10-fold slower *k*_obs_ rate than WT, 0.21 s^–1^ vs 2.4 s^–1^, respectively, suggesting that the S180R variant has slower presteady state kinetics (prior to, or during phosphodiester bond formation) than WT. In addition, S180R exhibits a 7.9-fold slower steady state rate of nucleotide incorporation (S180R *k*_ss_ = 0.058 s^–1^ and WT *k*_ss_ = 0.46 s^–1^), which suggests a slower rate of DNA or pyrophosphate product release.

**Figure 5:**
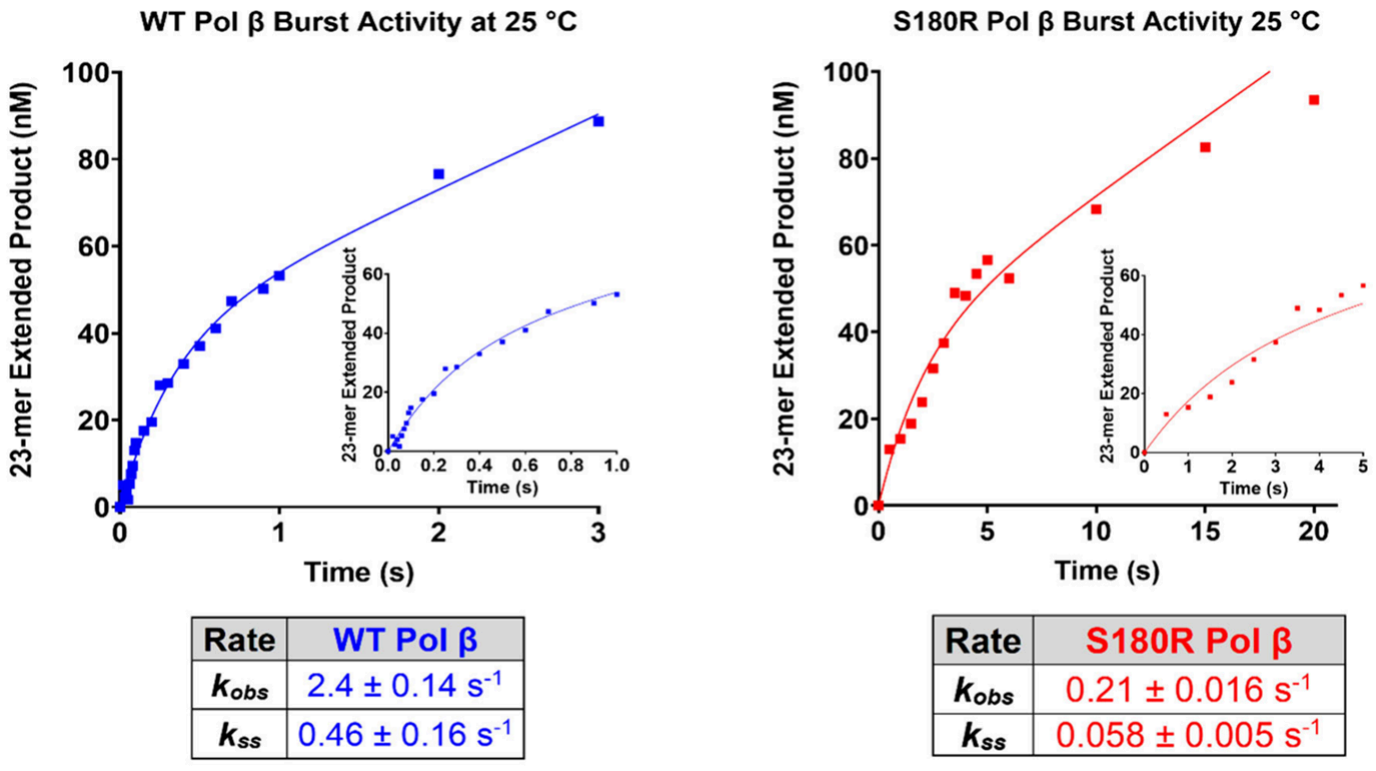
Biphasic burst kinetics demonstrate that S180R Pol β has a slower rate of burst as well as steady state compared to WT at 25 °C. The presteady state burst phase demonstrates a *k*_obs_ rate for S180R of 0.21 ± 0.016 s^–1^ compared to WT *k*_obs_ of 2.4 ± 0.14 s^–1^. Steady state kinetics for S180R show *k*_ss_ of 0.058 ± 0.005 s^–1^ whereas WT has a *k*_ss_ of 0.46 ± 0.16 s^–1^. Product formation was quantified and fit to the biphasic burst equation. Figures are representative experiments representing at least two biologic replicates, and data are reported ± SEM. Important to note the time scale difference on the *x*-axis.

### S180R Has a Slower Rate of Correct Nucleotide Incorporation

Single turnover kinetics were employed to examine the rate of nucleotide incorporation (*k*_pol_) for S180R relative to WT for single nucleotide gapped DNA with each of the four different templating bases. These experiments were performed to characterize substrate specificity given that Pol β fidelity can vary depending upon the specific nascent base pair.^34,52,54^ Given the location of the S180R residue mutation and the slow burst kinetics (slow *k*_obs_), we expected that the variant would exhibit a slower rate of nucleotide incorporation (*k*_pol_). Our data show for incorporation of dNTPs opposite all templating bases (C, G, T, A), the S180R variant has a slower *k*_pol_ rate (Table 1).

For dGTP coming in opposite C-template, the *k*_pol_ rate is 3-fold slower for S180R relative to WT (WT *k*_pol_ = 2.3 ± 0.33 s^–1^ and S180R *k*_pol_ = 0.82 ± 0.17 s^–1^). Similarly, for dCTP paired opposite G-template DNA, the S180R variant is 4-fold slower than WT as demonstrated by *k*_pol_ values of 0.92 ± 0.16 s^–1^ and 3.9 ± 0.14 s^–1^, respectively. For the dATP opposite T-template, again the S180R variant has a 3-fold slower *k*_pol_ rate than WT Pol β (WT *k*_pol_ = 3.0 ± 0.30 s^–1^ and S180R *k*_pol_ = 1.1 ± 0.15 s^–1^). Finally, for the dTTP opposite A-template, we find that the S180R variant is 6-fold slower than WT Pol β for its rate of nucleotide incorporation (WT *k*_pol_ = 6.7 ± 1.30 s^–1^ and S180R *k*_pol_ = 1.1 ± 0.14 s^–1^). These data support the conclusion that the S180R mutation within the dNTP binding pocket disrupts the rate of nucleotide incorporation universally, in all sequence contexts.

### S180R Binds Correct Nucleotides with a Lower Affinity Compared to WT

Single turnover kinetic data can be used to determine the *k*_pol_ rate, as discussed above, as well as the apparent dNTP binding affinity based upon *K*_m_ (defined here as apparent *K*_d(dNTP)_). Apparent *K*_d(dNTP)_ values for the S180R demonstrate that this variant binds to correct nucleotides with a lower affinity than WT for all templating bases (Table 1). For dCTP opposite the G-template and dGTP incoming opposite the C-template, we observe a dramatic 50-fold increase in the *K*_d(dNTP)_ values for the variant (G-template: WT *K*_d(dCTP)_ = 2.1 ± 0.06 μM and S180R *K*_d(dCTP)_ = 96 ± 13 μM; C-template: WT *K*_d(dGTP)_ = 1.0 ± 0.14 μM and S180R *K*_d(dGTP)_ = 58 ± 3.1 μM). Similarly, for T and A template correct single turnover kinetics, we also observe an increase in the *K*_d(dNTP)_ value for S180R. For A-template correct pairing there is a 50-fold difference in dTTP binding affinity (WT *K*_d(dTTP)_ = 7.4 ± 1.3 μM and S180R *K*_d(dTTP)_ = 405 ± 19 μM). For the T-template, WT has a *K*_d(dATP)_ of 0.31 ± 0.001 μM and S180R has a *K*_d(dATP)_ of 91 ± 8.4 μM, which represents a 290-fold difference in dATP binding affinity.

### S180R Misincorporates Incorrect dNTPs at Slower Rates Despite Having a Similar Binding Affinity As WT

Examination of the results for all templating bases reveals that the S180R variant incorporates incorrect dNTPs at a slower rate than WT (ranges from 4- to 47-fold slower, Table 1) suggesting that it is an inherent property of S180R. The *K*_d(dNTP)_ values are variable for incoming incorrect dNTPs, however, the degree of difference between WT and S180R is a subtle 1- to 2-fold difference. When contrasted with the dramatic loss of binding affinity of the correct nucleotides discussed above, the loss of fidelity of the S180R variant is driven by decreased binding affinity for the correct nucleotide.

For incorporation of incorrect dNTPs opposite the C-template, the S180R variant *k*_pol_ rates are slower than those of WT and incorrect nucleotide binding is comparable to WT values (Table 1). For C:dCTP (template:nucleotide), S180R’s *k*_pol_ is 13-fold slower than WT and the *K*_d(dCTP)_ value for S180R is only 1.6-fold greater than WT. There is a similar pattern for S180R having a slower nucleotide incorporation rate with dATP and dTTP incoming opposite C-template. For dATP, S180R has a *k*_pol_ that is 15-fold slower than that of WT and for dTTP the *k*_pol_ is 100-fold slower than that of WT. For dATP and dTTP opposite template C, WT and S180R have comparable *K*_d(dATP)_ and *K*_d(dTTP)_ values.

Opposite G-template S180R *k*_pol_ rates for incorporation of incorrect dNTPs are also slower than WT and *K*_d(dNTP)_ values are variable, but not dramatically different than WT (Table 1). With G:dATP, S180R is about 4-fold slower to incorporate dATP compared to WT. The nucleotide binding affinity of S180R for dATP is about 2.2-fold less than WT. For G:dTTP, S180R is about 47-fold slower to incorporate dTTP than WT, and it appears that S180R binds dTTP about 1.7-times more tightly than WT. Interestingly, for G:dGTP, we were unable to detect any product formation for S180R up to the maximum of 5000 μM dGTP concentration. While WT is generally slower at inserting dGTP opposite the G-template relative to other incorrect bases opposite the G-template, it appears that S180R has a significant defect for the insertion of dGTP opposite the G-template.

T-template incorrect kinetics show that S180R remains slower than WT for nucleotide incorporation, and S180R has a similar affinity for incorrect nucleotides relative to WT (Table 1). S180R is 21-fold slower than WT at incorporating dCTP opposite T-template, and binds dCTP 1.7-times more tightly. For incorporation of dGTP opposite T-template, S180R is 46-fold slower than WT and binds dGTP about 2.2-fold more tightly. For dTTP opposite T-template, S180R is 21-fold slower than WT at incorporating dTTP, and binds dTTP 1.8-times more tightly.

A-template studies show that S180R has a slow rate of incorrect nucleotide incorporation and similar nucleotide binding affinities (Table 1). With A:dCTP, S180R is 14-times slower and binds dCTP in this context with an affinity similar to that of WT. For dATP incoming opposite A-template, S180R is 8.8-fold slower and binds dATP with a similar affinity. Similar to what was described above for the purine–purine (G-G) pairing, S180R does not incorporate dGTP opposite an A-template even under conditions with the highest concentrations and time points.

### The Decreased Fidelity of S180R Is Due to a Lack of Nucleotide Discrimination at the Level of *K*_d_

Using calculations described in materials and methods to determine the discrimination values for *k*_pol_ and *K*_d(dNTP)_, we observe that S180R consistently demonstrates that it is unable to discriminate between correct and incorrect nucleotides, as evidenced by the loss of discrimination at the level of *K*_d(dNTP)_ (Table 1, Column 6). However, the variant also consistently gains the ability to discriminate against incorrect nucleotides at the level of *k*_pol_ (Table 1, Column 5). This is also evidenced by the correct single turnover data, demonstrating that S180R has a slower phenotype than WT, and S180R becomes even slower for incorrect pairings.

S180R’s relative inability to selectively bind correct nucleotides over incorrect nucleotides appears to outweigh its slower polymerization phenotype and is predominantly responsible for its low fidelity. Calculations of nucleotide incorporation efficiencies demonstrate that S180R is less efficient overall compared to WT for both correct and incorrect nucleotide incorporation (Table 1, Column 7). Fidelity calculations show that S180R has a lower fidelity relative to WT (Table 1, Column 8), as represented by relative X-fold values greater than 1. With the exception of incorrect nucleotides that could not be measured (G:dGTP and A:dGTP), we see the relative X-fold values demonstrate that S180R has a 2- to 69-fold lower fidelity than WT. For C-template, S180R’s fidelity loss is the most moderate ranging from 2- to 9-fold that of WT, and G-template demonstrates a loss of fidelity ranging from 7- to 19-fold. However, in comparison, for T-template DNA, S180R’s fidelity loss much greater ranging from 40- to 69-fold lower fidelity than WT. Similarly for A-template, fidelity loss ranges from 27- to 31-fold. Regardless of the templating base, the S180R variant demonstrates a universally lower fidelity than WT due to its loss of discrimination at the level of *K*_d(dNTP)_. However, it is interesting that the S180R variant has greater loss of fidelity for A- and T-templates, both of which can only stabilize the incoming base of the nucleotide with two hydrogen bonds. This suggests that for the templating bases, having only two stabilizing hydrogen bonds may contribute to the S180R variant’s lower fidelity.

### Binary Complex Crystal Structures Show a More Rigid Structure for S180R and Shifts in the Templating Base

For both the WT and the S180R variant, all four templating base options were crystallized, and structures refined. Isomorphous difference maps for the templating options were investigated for common peaks, positive and negative. Consistent though minor negative (red) difference peaks were observed within the region surrounding the R180 mutation at the sidechains of R149 and E186, the backbone of S187 most likely indicated an increase in flexibility or disorder. The most striking differences were observed at the templating base position, the primer terminus and R254 (Figure 6), whose side chain forms a salt bridge to D256 which coordinates the catalytic Mg^2+^ metal ion. The R254 side chain shows a strong positive isomorphous difference density (blue mesh *F*_obs(S180R)_–*F*_obs(WT)_) for the dA, dT, and dG and weak for the dC templating positions relative to WT. This was not through a change in position, as its position is identical for WT in each case but likely a decrease in flexibility. This becomes evident in calculating the B-factor (thermal factor) ratio of the side chain relative to the main chain for each structure. For WT, the ratio of side chain B-factor to main chain B-factor is 1.42, 1.29, 1.65, and 1.32 for dA, dG, dT, and dC templating, respectively. The number >1 indicates a greater degree of flexibility for the side chain relative to the main chain, as is quite common. However, for S180R the ratios were 1.03, 1.04, 1.11, and 0.93, respectively, indicating minimal difference between side chain and main chain flexibility.

**Figure 6:**
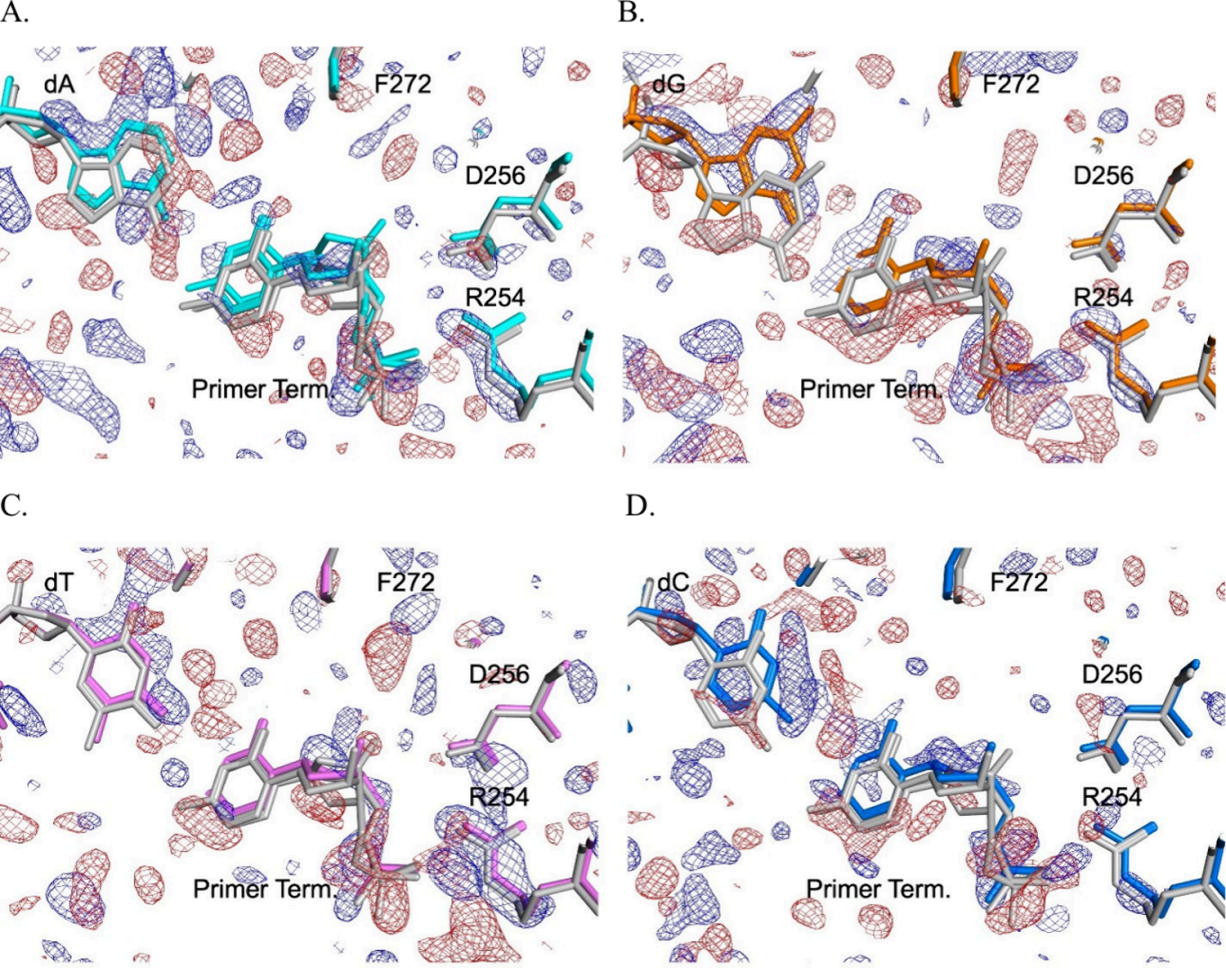
Isomorphous difference Fourier analysis of the S180R variant compared to that of WT of DNA pol β binary complexes. Shown are the superpositions of the 4 templating position options for S180R relative to WT (gray). Isomorphous difference Fourier maps^50^ contoured at 2.5 σ calculated as *F*_o(S180R)_ – *F*_o(WT)_ with blue difference density indicating entities or positions gained in the variant compared to WT while red difference density indicates entities or positions lost in the variant relative to WT. Complexes shown are for templating position dA (A, cyan), dG (B, orange), dT (C, violet), and dC (D, blue). In this figure and throughout the text, blue and red are used to represent positive and negative electron density, respectively, instead of the customary green and red. This choice was made to improve the visibility for individuals with color vision deficiencies.

Neighboring the R254 side chain, difference maps show disruption of the primer terminus at the phosphodiester linkage to the −1 position of the primer strand and the position of the deoxyribose ring, of which the 3′-OH must also coordinate the catalytic metal. The incoming nucleotide in a ternary complex is typically needed to assemble the active site and provide the nonbridging oxygen and reposition D192 as the additional ligands for the catalytic Mg^2+^. Consistently observed in the isomorphous difference maps were a peak–hole pair (blue and red) at the deoxyribose. During model refinement, it was evident that the WT complexes universally had a dual conformation of the primer terminus (conformers 1 and 2) while that was the case for S180R only with dA in the templating position and weakly at that. The two conformations do not differ in sugar pucker but geometry changes at the phosphodiester linkage to the −1 position in the primer strand as determined using x3dna^55^ (Figure S1). While additional primer terminus rearrangement occurs in transition to the precatalytic ternary state^40^ or during the time-resolved reaction structures,^56^ conformation 2 points the 3′-OH toward the Mg^2+^ coordination site and most resembles transition to the precatalytic complex while conformation 1 does not. The dG, dT, and dC binary complexes for S180R favor only conformation 2 indicating a restriction in dynamics for the variant. In comparison, the WT binary complexes favor a split between conformations 1 and 2 of the primer terminus. The templating base also showed significant peaks in the difference map, and all but the dT produced a refined model with the base rotating toward the fingers domain and away from the Watson–Crick interface. This was most pronounced for the dG and dC (Figure 6). Rotation of the templating base was additionally accompanied by changes in the water coordination for complexes dC, dA, or dG (Figure 7).

**Figure 7:**
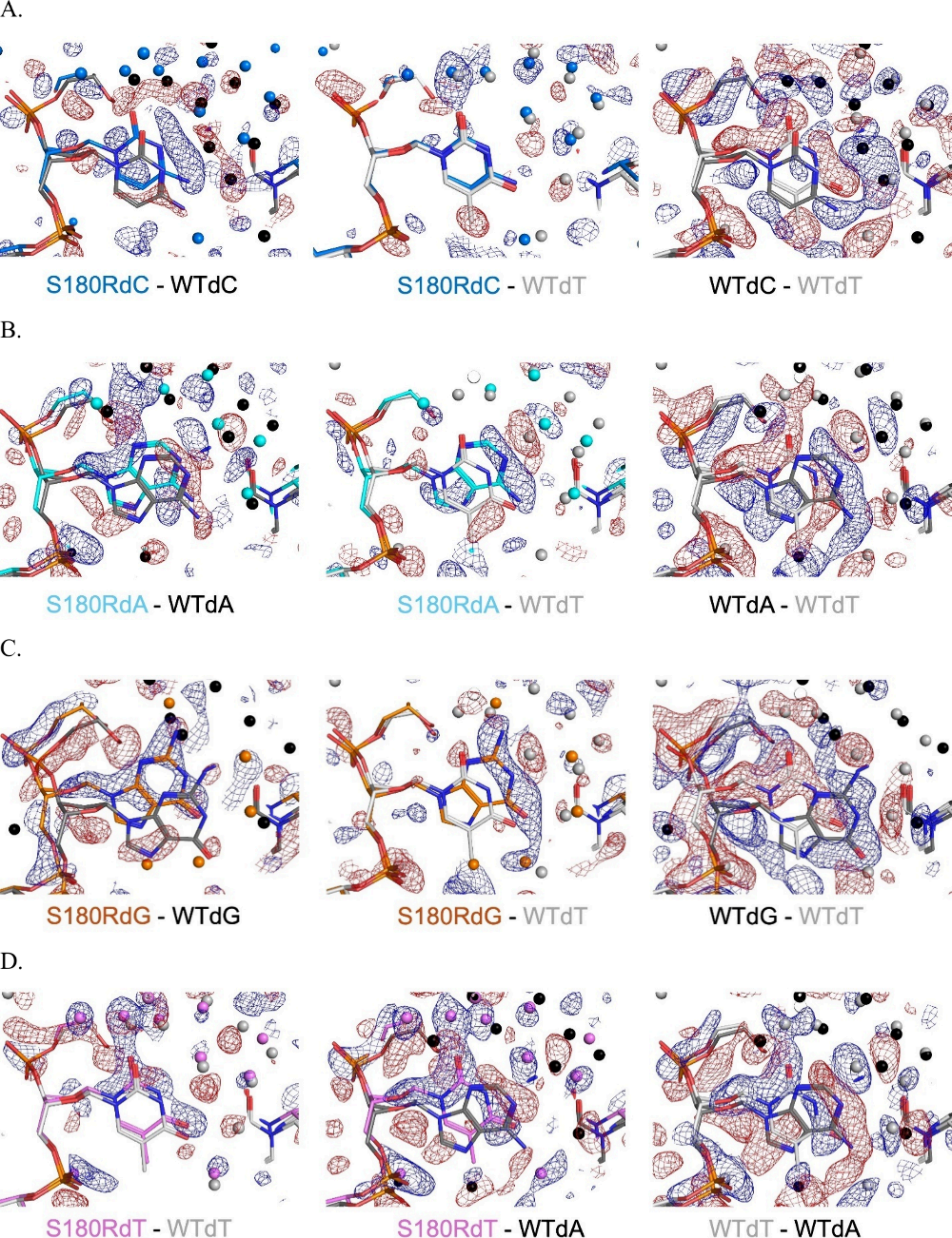
Binary complex isomorphous differences for S180R compared with the WT for the four templating positions. For each panel, the isomorphous difference Fourier maps of *F*_obs_–*F*_obs_ are +3 σ (blue) or −3 σ (red) for data set 1 minus data set 2 as indicated. The S180R variant showed consistent misalignment of the templating base relative to WT (left column for sets A, B, C, D) while the S180R variant showed the least difference when compared to the WT dT template (middle column for sets A, B, C). Spheres indicate water and are color coded according to the complex label. The right column shows WT–WT templating base differences for direct comparison to the middle column, S180R–WT.

### Ternary Complex Structures Demonstrate Disrupted Nucleotide Coordinating Residues

Like the binary complexes, all four templating base options in complex with cognate dNTP were determined and minimal global structural deviation was observed for the S180R variant relative to WT. However, the isomorphous difference maps provided compelling differences for the variant with stable placement of the R180 (Figure 8 blue mesh) side chain juxtaposed to the γ-phosphate of the incoming nucleotide and a strongly correlated loss of the S180 position (Figure 8 red mesh). Strong negative difference peaks were observed for the R149 side chain in all four cognate complexes (Figure S2) along with a complementary positive difference peak indicating a position change for R149 in the variant relative to WT structure. All incoming nucleotides show positive difference peaks surrounding the nonbridging oxygens of the γ-phosphate. Initially suggestive of the γ-phosphate being drawn toward the R180 imido group, a difference in the bond lengths coordinating the nucleotidyl metal was anticipated. However, octahedral coordination of both the nucleotidyl Mg^2+^ and catalytic Mg^2+^ was retained with only the 3′-OH distance to the catalytic metal for the dA:dUmpNpp complexes of both WT and S180R deviating from standard length (Figure S3). As observed for the varying template base positions in the binary complexes, the coordinated water structure was significantly altered for the variant. This included the loss of three waters (bridging S180 to R183, R149 to S180, and R149 to the γ-phosphate) and a shift for the water pair adjacent to the conformationally shifted E186.

**Figure 8:**
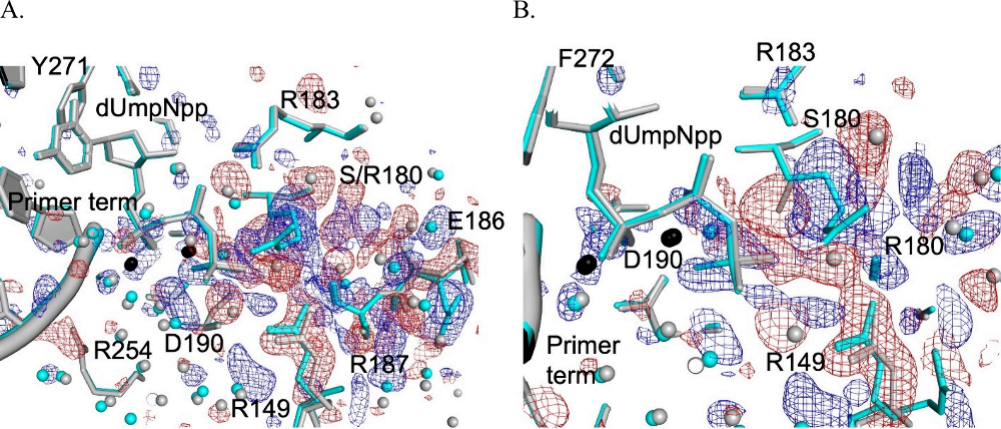
Isomorphous difference Fourier analysis of DNA pol β for the dA:dUmpNpp ternary complex for variant S180R (cyan) and WT (gray) contoured at ±3 σ calculated as *F*_o(S180R)_ – *F*_o(WT)_. The single complex is shown in two orientations (A and B) for clarity while the dG:dCmpCpp, dT:dAmpCpp, and dC:dGmpCpp can be found in Figure S2. Blue difference density indicates entities or positions gained in the variant compared to WT while red difference density indicates entities or positions lost in the variant relative to WT. The black spheres are the catalytic and nucleotidyl Mg^2+^ ions while the light spheres (gray/cyan) are water molecules.

### Data Correlation and Global Comparisons Demonstrate That S180R Has Decreased Fingers Domain Dynamic Movements in the Binary State

All S180R templating structures were reasonably isomorphous with their respective WT reference structure, as determined by the overall *R*_merge_ % or correlation coefficient (Figure 9) between the two data sets. This indicates minimal global structural difference between the S180R variant and the WT and is important as the lyase domain and fingers domain experience significant conformational changes between enzymatic states. The correlation plots also corroborate the isomorphous difference map interpretation where the templating dT (Figure 9A, red) showed the highest correlation between S180R and WT (Figures 6C and 7D) and smallest differences in the map. Importantly, this was also the case for WT dT relative to S180R dC where the structure, difference maps, and data correlation showed the highest agreement. The S180R dA and dG templates, like the trends in the difference maps, also showed a greater data correlation to the WT dT template than their respective WT equivalents. While final refined models can have error limitations and such small differences between variant and WT, the S180R analysis suggests that diffraction data alone can indicate the error-prone nature of some variants. A much higher correlation coefficient for *F*_obs_ was observed for the equivalent ternary complexes relative to binary complexes indicating fewer differences between WT and variant in the presence of dNTP and Mg^2+^.

**Figure 9:**
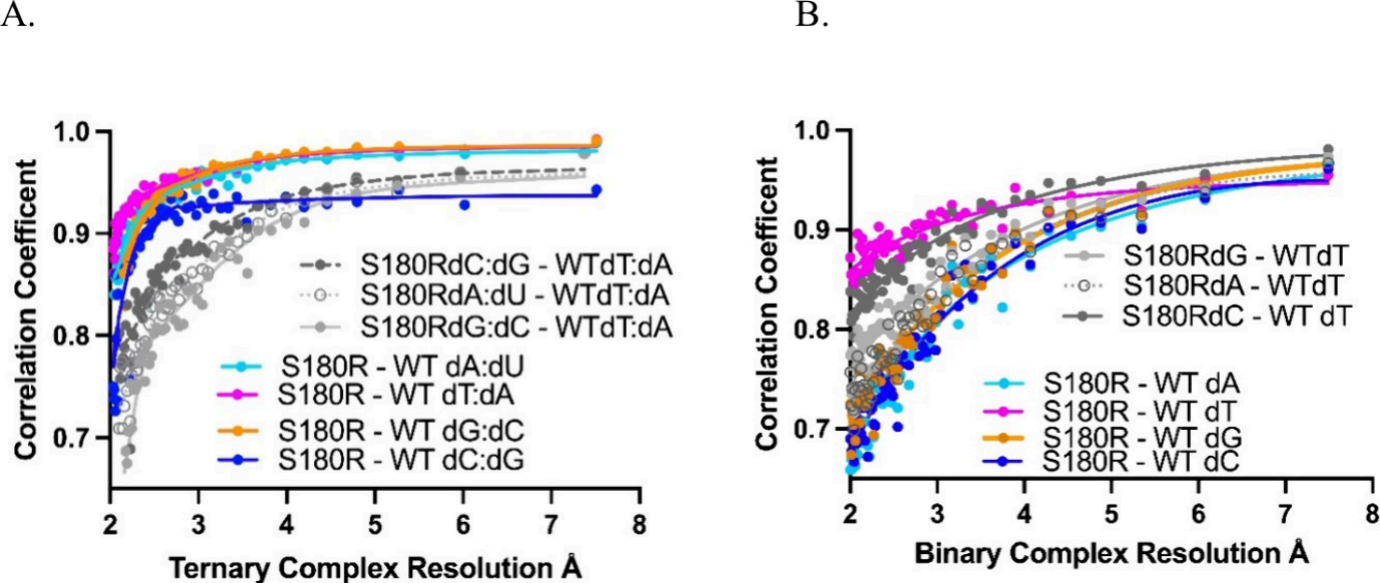
Diffraction data correlation coefficients (CC) between WT and S180R Pol β for equivalent templating position complexes (dT in magenta, dG in orange, dA in cyan, and dC in blue). The plot shows the degree of agreement between two particular data sets independent of model indicating global structural agreement across the resolution range with a CC of 1.0 being identity. A, binary complexes; B, ternary complexes. The correlation is determined via isomorphous difference calculation using *F*_obs_ within Phenix.^49^ For each plot, the correlations between WT dT and S180R dG, dC, and dA complexes are shown in gray. In the binary state, the gray plots (WT dT vs S180R non-dT templates) show better correlation than presumed identical complexes with WT dT and S180R dC (dark gray) having the second highest CC of the series. This is not the case for the ternary comparison where the gray plots all show the lowest correlation as would be expected for nonidentical complexes.

Plots of the refined B-factors for the polymerase were generated and indicate a dynamic difference between the WT and S180R fingers domain in the binary state (Figure 10). The B-factors are a thermal correction to account for model deviation or flexibility observed relative to the data. In the binary complex, fingers are more flexible then get locked down upon binding the correct incoming dNTP in a ternary complex.^57^ Conformational dynamics of the fingers domain have long been implied for selectivity for the incoming nucleotide as the step repositions key residues in the active site as described in ref 58 and references therein. For all templating positions, S180R showed a compressed B-factor range between the fingers and the rest of the protein (lower dynamic range) in the binary state. This was independent of the templating base as all four plots superimpose within the WT or S180R series (Figure 10A,B). The median B-factors per lyase, thumb, palm, and fingers domains were 15.4, 14.9, 18.7, and 37.7 Å^2^ for WT and 23.3, 22.1, 26.8, and 39.8 Å^2^ for S180R, respectively. Calculating the fold increase in B-factor of the fingers domain relative to the lyase, thumb, and palm shows the WT as 2.44, 2.53, and 2.01 but S180R as 1.71, 1.81, and 1.48 Å^2^, respectively. This was not observed in the ternary state.

**Figure 10:**
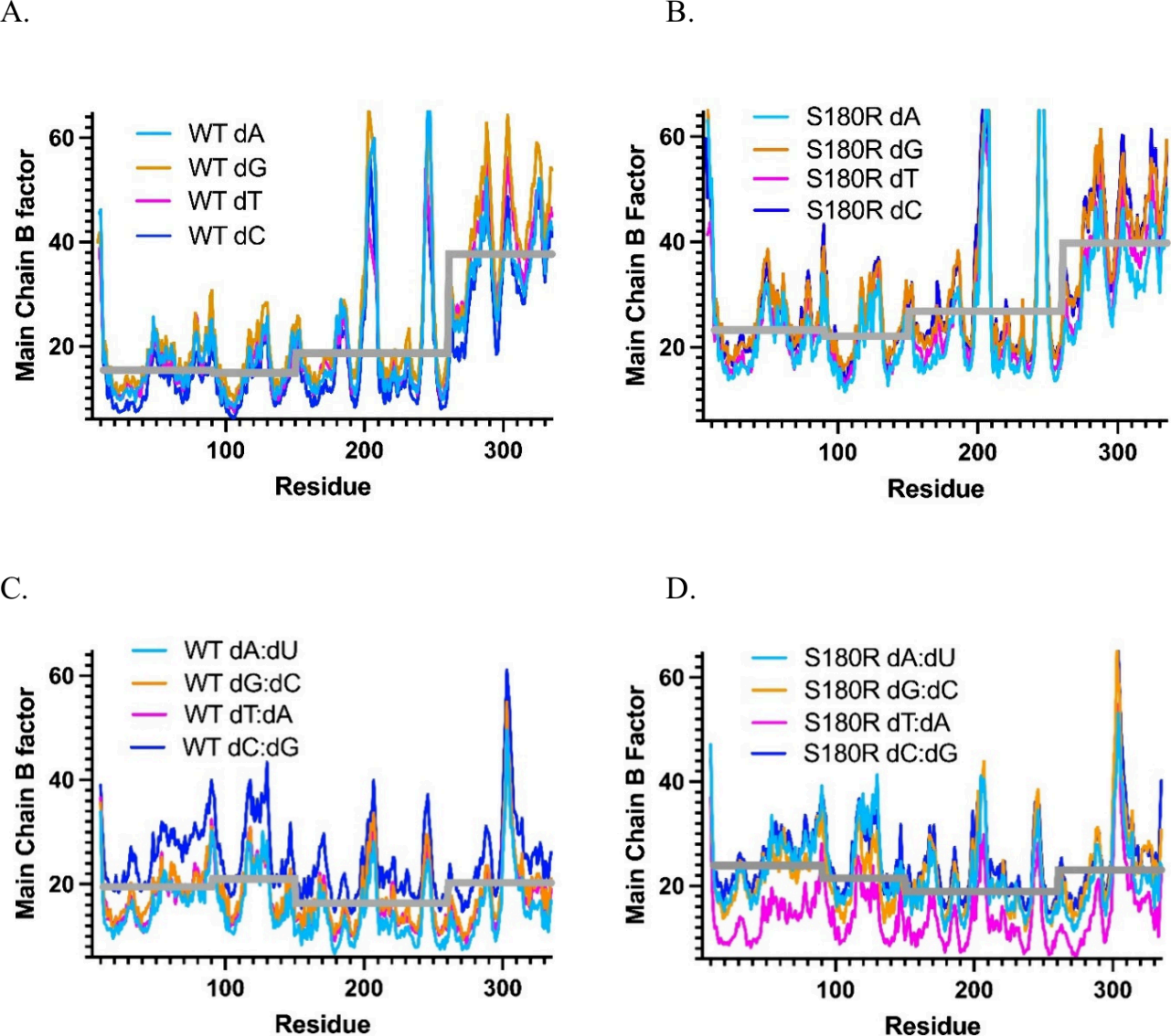
Main chain B-factors (Å^2^) for the binary complexes of WT (A), S180R (B) and the ternary complexes of WT (C), S180R (D), with templates labeled (dT in magenta, dG in orange, dA in cyan, dC in blue). The approximate structural domain definitions for Pol β are as follows: residues 1–90, lyase; residues 91–150, thumb; residues 151–260, palm; residue 261–335, fingers. The gray line on each plot represents the B-factor (Å^2^) by domain averaged from all 4 structures in the series. The highly flexible loops are 200–210, 240–250, and 300–310 and plots are on identical scales. The fingers domain in the absence of incoming nucleotide will be more flexible (higher B-factor), while the other domains contact the DNA duplex. Binary S180R complexes (B) have a clear suppression of the dynamic difference between the fingers domain and remaining protein compared to that of WT (A) that is not observed for ternary complexes (C, D).

## Discussion

Here we characterize the S180R human germline variant of Pol β (rs1585898410) and show that this novel human germline variant has an inherently low fidelity of DNA synthesis and structural alterations that are distant from the mutation. This variant has a mutation of residue S180 which coordinates with the γ-phosphate of the incoming dNTP, and introduces a bulky and positively charged arginine within the active site of Pol β (Figure 8). Based on our kinetic studies, fidelity loss in the S180R variant appears to be influenced by a loss of nucleotide discrimination (discrimination *K*_d_) as opposed to the effect of the rate of nucleotide incorporation (discrimination *k*_pol_) for all four templating bases (Table 1). Our structural data reveal disruption in the binary state fingers domain that could be contributing to the low fidelity of this variant as well as ternary disruption of nucleotide coordinating residues likely resulting in the low nucleotide binding affinity (*K*_d_) of the S180R variant.

Upon binding a dNTP, Pol β undergoes closure of its fingers domain, which is thought to align catalytic residues to facilitate nucleotidyl transferase activity. Interestingly, the S180R variant has decreased fingers domain flexibility in the open binary state, as evidenced the compressed B-factor range between the fingers and the rest of the S180R protein (Figure 10). The fingers transition from open to closed is coordinated with side chain rearrangements differentially stabilized via unique interactions within either the open or closed state. It has been shown that these rearrangements during the fingers closing and noncovalent steps prior to nucleotide incorporation are critical for maintaining fidelity.^22,59^ Recently, another low fidelity variant of Pol β (I260M) has been shown to have a collapsed fingers domain which is thought to be the basis for its low fidelity.^60^ There is growing evidence that Pol β’s binary structure is influential in maintaining fidelity, likely due to residue positioning for dNTP selection in preparation for nucleotidyl transfer.^32,60^

Structurally, our data show that in the binary state the S180R variant has disruption of the neighboring primer terminus and 3′OH in the setting of a more rigid fingers domain and R254 side chain (Figures 6 and 7). The more rigid fingers domain structure and disruption of active site water molecules is likely a major contributor to altered primer positioning and limited conformations seen in S180R. However, residue R254 is one of three residues known to position the primer terminus,^61,62^ and it stabilizes the 3′OH during proton transfer.^63^ It is also possible that the R254 side chain’s rigidity is contributing to the perturbed primer positioning either through its direct interactions or indirectly through disruption of its coordinating active site residue D256. Notably, the primer terminus position in the S180R binary structure is almost completely in conformation 2 which more closely resembles the ternary structure (Figure S1). This could represent in part the basis of the low fidelity for the S180R variant if this second conformation favors ternary formation for both correct and incorrect nucleotides once they are within the active site. In this study, we show that the S180R variant has decreased fingers flexibility in the binary state. We propose that this loss of finger flexibility is most likely causing disruption of the primer terminus position in the binary state and contributing to the S180R variant’s low fidelity. Importantly, both disruptions occur at the binary state, prior to dNTP binding, which supports the growing evidence that the binary structure can be influential to Pol β fidelity.

The ternary state of the S180R variant appears similar to WT, except for the disruption proximal to the γ-phosphate-coordinating water molecules and dual conformation and/or decreased occupancy of the R149 residue for all correct nucleotide pairings (Figure 8). This disruption of γ-phosphate coordination could decrease the stability of the nucleotide within the binding pocket and is the likely cause for the low dNTP binding affinity in this variant (*K*_d_). There is evidence that subtle nucleotide rearrangements occur through coordinating residues including S180 and R149.^56^ Kraynov et al.^27^ looked at the effect of alanine substitutions within these residues (data summarized in Table S1). When comparing the S180R variant kinetic data to the published data, we see that the S180R mutation has a more dramatic effect on the correct nucleotide binding affinity (*K*_d_) than either S180A or R149A alone. Since the S180R mutation displaces residue R149 due to electrostatic repulsion, the loss of two γ-phosphate-coordinating residues may be driving the more dramatic *K*_d_ effect. This suggests that γ-phosphate coordination by both S180 and R149 have potential synergism for coordinating correct nucleotide binding and providing stability of the nucleotide within the active site. Interestingly, the S180A substitution results in a greater decrease in *k*_pol_ relative to that of S180R, while R149A had no effect on *k*_pol_. This suggests that the S180 residue is more important for polymerase activity, and the R149 residue only contributes to nucleotide binding. Our research emphasizes the importance of these two γ-phosphate interacting residues (S180 and R149) as nucleotide stabilizing residues in the ternary state.

The steady state rate (*k*_ss_) under burst conditions is known to be a function of enzyme turnover (product release and rebinding of a new DNA substrate). Since WT and S180R exhibit similar DNA binding affinities (Figure 3), but S180R has a much slower rate at steady state (Figure 5), it is likely that the S180R variant exhibits a slower rate of pyrophosphate product release compared to that of WT. The S180R mutation places a positively charged residue in proximity to the γ-phosphate, which could allow for tight binding of pyrophosphate product following phosphodiester bond formation. It is thought that S180 may play a role in orienting the leaving group at the transition state,^27^ and this may be disrupted in S180R. The structural data also show disruption of coordinating waters that form hydrogen bonds stabilizing the γ phosphate groups in the ternary state, which could also be disrupted in the postcatalytic state and contribute to preventing pyrophosphate product release. To date, studying product release remains challenging in Pol β through both kinetic assays and crystal structures. Given that within the crystal structures, the fingers domain does not open after correct insertion and pyrophosphate product remains bound,^64^ it is difficult to interpret product release from structural data. If product release is delayed in this variant, it could be significant within the cellular environment, as it would inhibit protein turnover and eventually lead to decreased efficiency of single nucleotide gap filling *in vivo*.

Our data also show that the melting temperature of the S180R variant is lower than that of WT (Figure 4) indicating that there may be structural instability in the apoenzyme (unbound) form as a result of the introduction of the S180R mutation in proximity to residues R149 and R183, causing repulsion and lack of stability in the apoenzyme, which would then be alleviated in the binary and ternary states. As circular dichroism is not meant to be predictive of structural stability within a dynamic cellular environment, it would remain important to understand how this low fidelity variant functions within cells. Increased temperatures up to that of a human body temperature of 37 °C could present new challenges for the S180R variant. First, it could cause complete unfolding of the protein as suggested by our T_m_ studies, which would result in only one functional copy of Pol β. It is known that Pol β haploinsufficiency increases mortality and cancer risk in mice,^65^ so this could also be driving increased cancer formation in these patients. However, in the dynamic cellular environment, with chaperone proteins, it is possible that this variant is folded at higher temperatures but may have increased flexibility of the fingers’ domain due to the impact of temperature on dynamics or structural stability. As crystal structures can predict but do not determine the true dynamics of protein movement given their static state, the examination of protein dynamics and fingers movements could be a valuable future direction to further characterize this variant.

## Conclusion

In this study, we identify and characterize the S180R variant (rs1585898410) as a rare human germline mutation of DNA polymerase β with compromised nucleotide selectivity and fidelity. Through integrated kinetic and structural analyses, we show that S180R exhibits a universal low-fidelity phenotype driven primarily by a loss of nucleotide discrimination at the level of dNTP binding affinity rather than the catalytic rate, despite an overall slowing of nucleotide incorporation. Structural data reveal that this defect is the result of altered binary state dynamics, including reduced fingers domain flexibility and perturbed primer terminus positioning. Together, these findings highlight the importance of the binary state for Pol β fidelity and support an emerging paradigm in which subtle perturbations to the binary state dynamics of Pol β can drive a loss of fidelity. We speculate that this human germline variant has the potential to induce mutations and genomic instability within human cells through low fidelity or stalled DNA repair. This germline variant could also have implications for cancer development in populations that harbor this genetic mutation. Overall, this work underscores the need for future cellular and *in vivo* studies to define the biological consequences of the S180R Pol β germline variant on DNA repair capacity and cancer susceptibility.

## Supplementary Material



## Notes

A table which compares our data with data published on relevant Pol β mutations (Table S1), refined structure of the dual conformation of the primer terminus (Figure S1), isomorphous difference maps for the ternary complexes (S2), and octahedral coordination distances (S3), a representative single turnover (STN) plot of product formation over time (S4) with correct STN rates plotted (S5) and incorrect STN rates plotted (S6–S9) (PDF)

## Notes

The S180R atomic coordinates and structure factors have been deposited to the Protein Data Bank at rcsb.org with PDB ID 9Y1D, 9Y1E, 9Y1F, 9Y1G for the binary and PDB ID 9Y1H, 9Y1I, 9Y1J, 9Y1K for the ternary. The WT atomic coordinates and structure factors have been deposited to the Protein Data Bank at rcsb.org with PDB ID 9Y15, 9Y16, 9Y17, 9Y18 for the binary and PDB ID 9Y19, 9Y1A, 9Y1B, 9Y1C for the ternary complexes.
