# Supplementary material for: The S180R Human Germline Variant of DNA Polymerase β Is a Low Fidelity Enzyme with Reduced Flexibility of the Fingers Domain
Source: Biochemistry. 2026 Jan 12;65(3):270–83. doi: 10.1021/acs.biochem.5c00628 (PMC13105415; doi:10.1021/acs.biochem.5c00628)
Supplement: Supplementary file 1 [file bi5c00628_si_001.pdf]

Supplementary Information:

Manuscript Title: The S180R human germline variant of DNA polymerase β is a low fidelity enzyme with reduced flexibility of the fingers domain

Authors: *Danielle L. Sawyer[1], Brian E. Eckenroth[2*], Cristian Chavira[1], Khadijeh Alnajjar[1], John P. Hanley[2], Julie A. Dragon[2], Sylvie Doublié[2], and Joann B. Sweasy[3*].*

[1]Department of Cell and Molecular Medicine, University of Arizona, 1515 N Cambell Avenue, Tucson, AZ 85724, United States.

[2]Department of Microbiology and Molecular Genetics, University of Vermont, Stafford Hall, 95 Carrigan Drive, Burlington, Vermont 05405, United States.

[3]Eppley Institute for Research in Cancer and Allied Diseases, Fred & Pamela Buffett Cancer Center, University of Nebraska Medical Center, Omaha, NE 68198, United States.

Co-corresponding Authors: Joann B. Sweasy (jsweasy@unmc.edu), Brian E. Eckenroth (brian.eckenroth@uvm.edu)

Supplementary Table 1: Kinetic parameter comparison of WT and variant Pol β from this study and Kraynov et al. *(27)*. Data from Kraynov et al. in blue, performed at 37 ˚C, and the data from this study shown in salmon (25 ˚C). Kraynov et al. demonstrate that R149A substitution affects the dNTP binding affinity but not the rate of polymerization. Whereas the S180A mutation affects both dNTP affinity and polymerization rate. The S180R mutation appears to affect K_d to a greater magnitude relative to the effect that is seen with the S180A or the R149A suggesting that the S180R mutation is more detrimental to nucleotide binding. Conversely, it appears that k_{pol} is less affected by the S180R mutation, suggesting that the addition of Arginine provides additional benefit for nucleotide incorporation.

K_d (μM)	WT	R149A	S180A	R183A	WT	S180R
A:dTTP	5.2	35	70	21	7.4	405
T:dATP	8.5	109	59	87	0.31	221
G:dCTP	1.9	12	x	5.9	2.1	96
	Kraynov (37 °C)			This study (25 °C)		
k_{pol} (s^{-1})	WT	R149A	S180A	R183A	WT	S180R
A:dTTP	21.9	24	1	0.9	6.7	1.1
T:dATP	36.3	21	0.57	0.83	3	0.94
G:dCTP	12.5	11	X	2.6	3.9	0.92

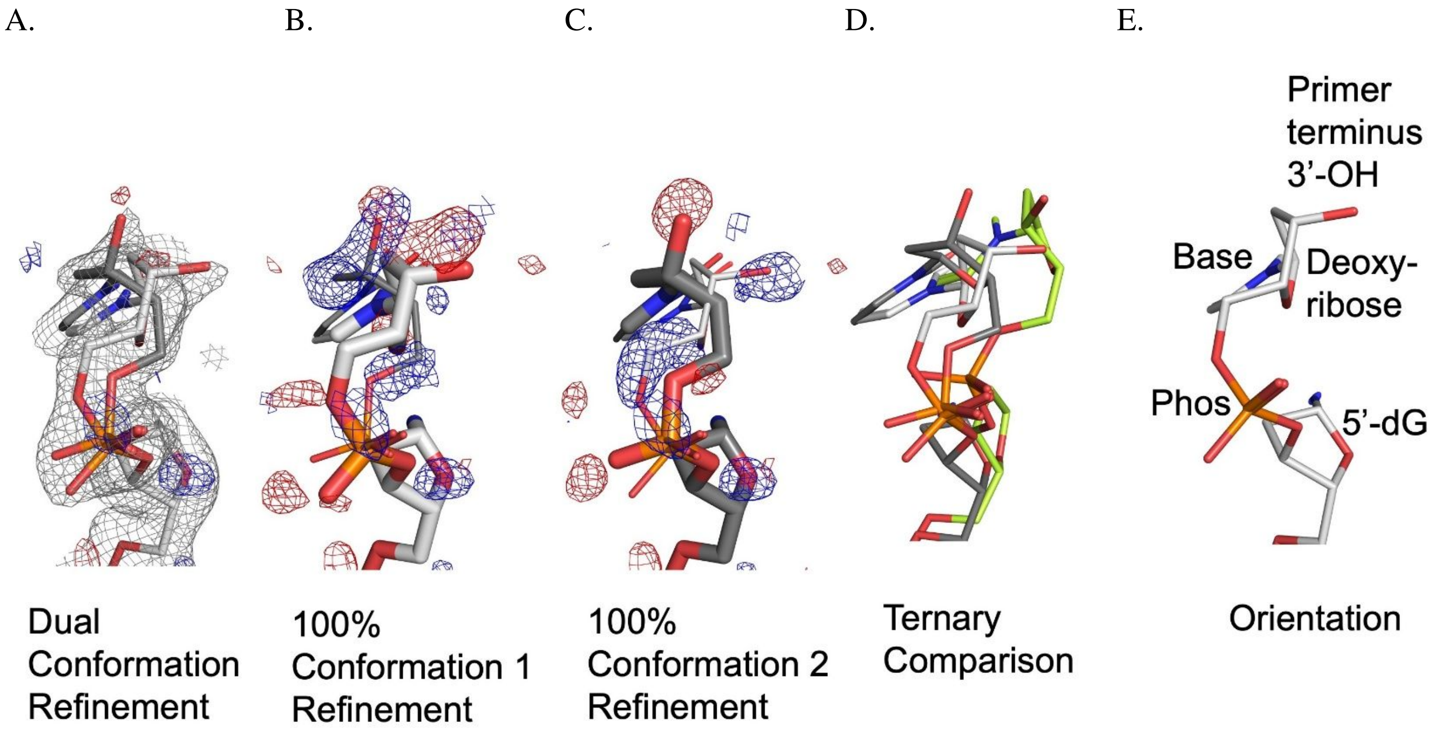

Supplemental Figure 1. Dual conformation refinement of primer terminus for WT Pol β. Shown are the refinement maps as 2Fo-Fc (grey mesh), and residual maps +/- 2.7 σ (blue and red mesh) for the WT binary structure containing dG in templating position (A). Refined with the light grey conformer #1 in the large stick format (B). Refined with the dark grey conformer #2 in the large stick format (D). Overlay (green model) of the WT ternary complex upon superposition of the palm domains (residues 150-260). Labeled primer terminus structure for orientation purposes (E).

A.

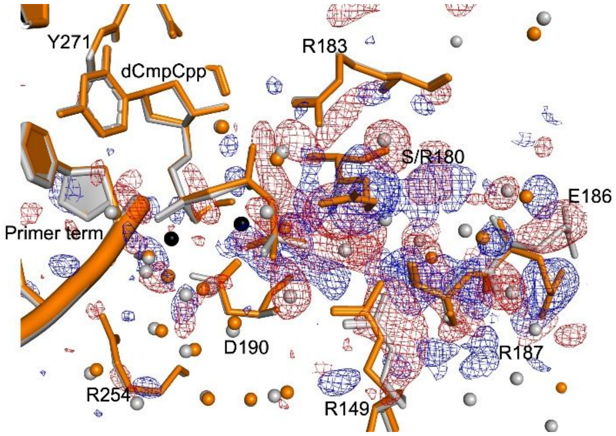
Y271
dCmpCpp
R183
S/R180
E186
Primer term
D190
R187
R254
R149

B.

S.4

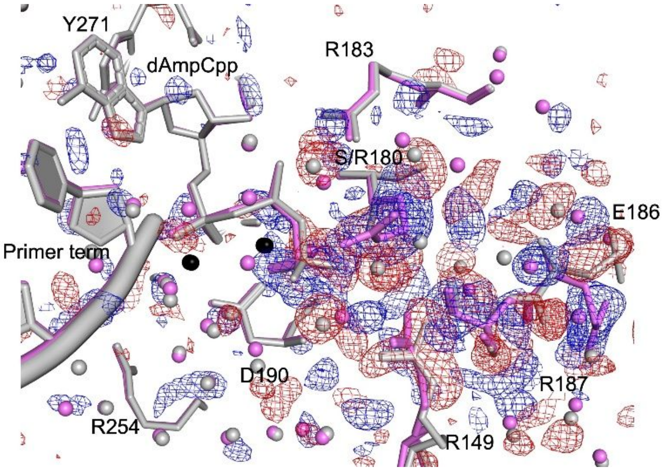

C.

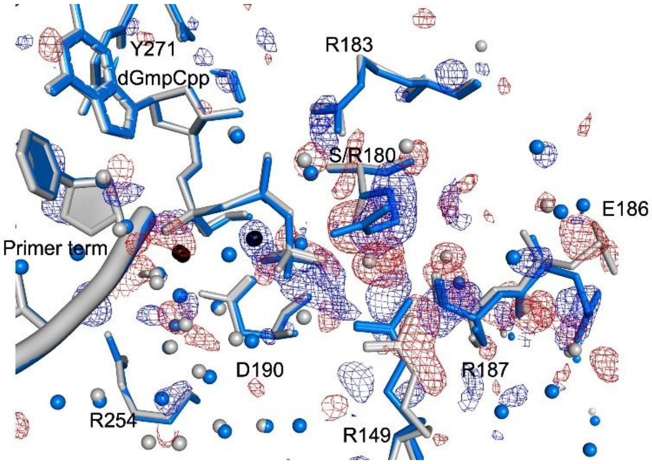

Supplemental Figure 2. Isomorphous difference Fourier analysis of DNA pol β for S180R (cyan) and WT (grey) ternary complexes contoured at +/- 3 σ calculated as $F_{o(S180R)} - F_{o(WT)}$. The S180R complexes shown are dG:dCmpCpp (A - orange), dT:dAmpCpp (B - violet) and dC:dGmpCpp (C - blue) with corresponding WT in grey. Blue difference density indicates entities or positions gained in the variant compared to WT while red difference density indicates entities or positions lost in the variant relative to WT.

A.

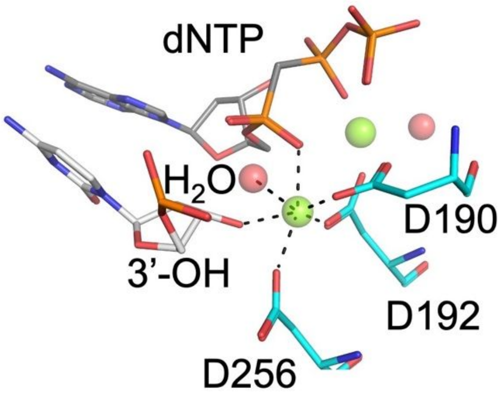

	D190	D192	D256	dNTP	3'-OH	H_2O
dAdU	2.1	2.2	2.2	2.3	2.8	2.2
dTdA	2.0	2.0	2.1	2.3	2.3	2.4
dGdC	2.0	2.0	2.1	2.2	2.2	2.1
dGdC	2.1	2.1	2.2	2.2	2.2	2.2
dAdU	2.0	2.0	2.1	2.3	2.6	2.3
dTdA	2.0	2.1	2.1	2.2	2.3	2.1
dGdC	2.0	2.1	2.0	2.2	2.2	2.0
dGdC	2.0	2.0	2.0	2.3	2.2	2.2

B.

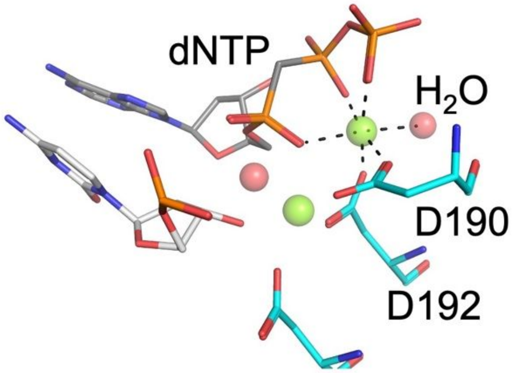

	D190	D192	dNTP	dNTP	dNTP	H_2O
dAdU	2.0	2.0	2.1	2.0	2.0	2.1
dTdA	2.0	2.1	2.1	2.1	2.0	2.1
dGdC	2.0	2.1	2.0	2.1	2.1	2.1
dGdC	2.0	2.2	2.2	2.1	2.0	2.2
dAdU	1.9	2.1	2.1	2.1	2.0	2.0
dTdA	2.0	2.0	2.1	2.0	2.1	2.1
dGdC	2.0	2.1	2.1	2.0	2.1	2.1
dGdC	2.0	2.0	2.1	2.0	2.1	2.1

Supplemental Figure 3. Metal octahedral coordination distances for ternary DNA pol β complexes. A. Shown are the coordination of the catalytic Mg^{2+}(left) and table of bond lengths (right), with WT in black font and S180R in blue using WT dT:dAmpCpp as the model. B. Shown are the coordination of the nucleotidyl Mg^{2+} (left) and table of bond lengths (right). All distances are in Ångstroms (Å).

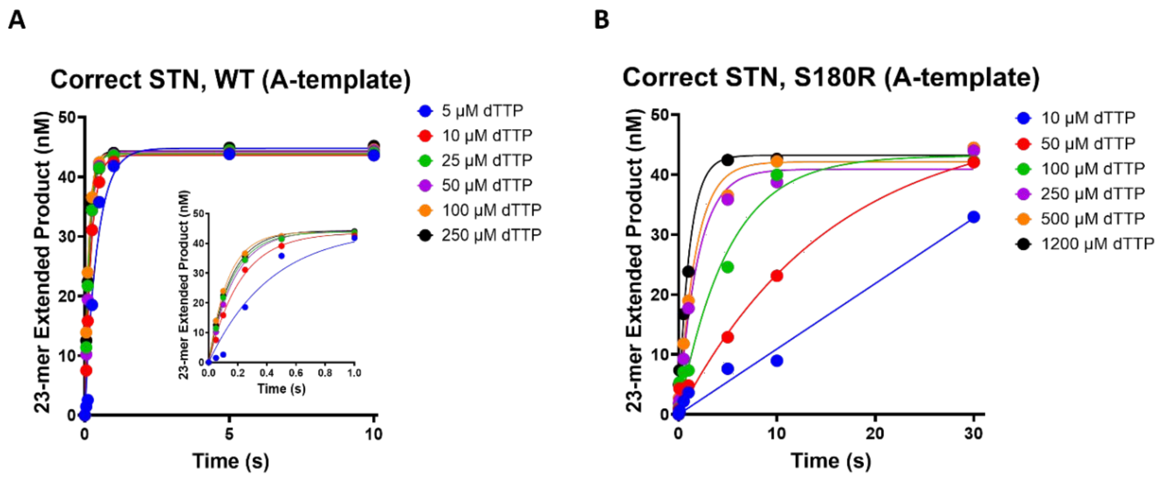

Supplementary Figure 4: Representative correct single turnover (STN) data at 25 ˚C demonstrating product formation over time for WT (A) vs S180R variant (B) at increasing concentrations of dTTP substrate. This data was used to calculate rates of nucleotide incorporation at each dNTP concentration, which was then plotted as in Supplementary Figure 5.

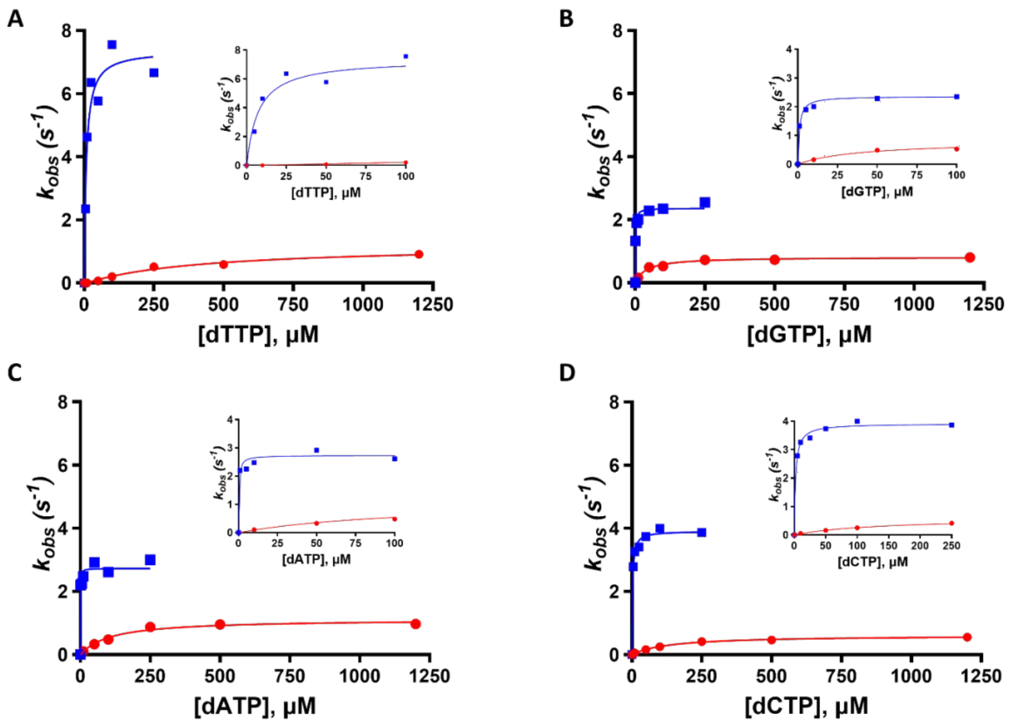

Supplementary Figure 5: Correct single turnover data for each DNA template, for WT (blue) and S180R (red) demonstrate that S180R is a slow variant under all sequence contexts, and with a decreased dNTP apparent dissociation constant ($K_{d(dNTP)}$). A-template (A), C-template (B), T-template (C) and G-template (D) data shown. Product formation over time was used to calculate a k_{obs} rate for each dNTP concentration (see representative panel in Supplementary figure 4). K_{obs} *rate*s were then used to estimate the k_{pol} rates which are reported as mean value ± standard error in Table 1. The apparent dissociation constant for dNTP ($K_{d(dNTP)}$) was then estimated from the data and reported as mean value ± standard error in Table 1. Data shown are representative plots, with a minimum sample size of two biological replicates per condition.

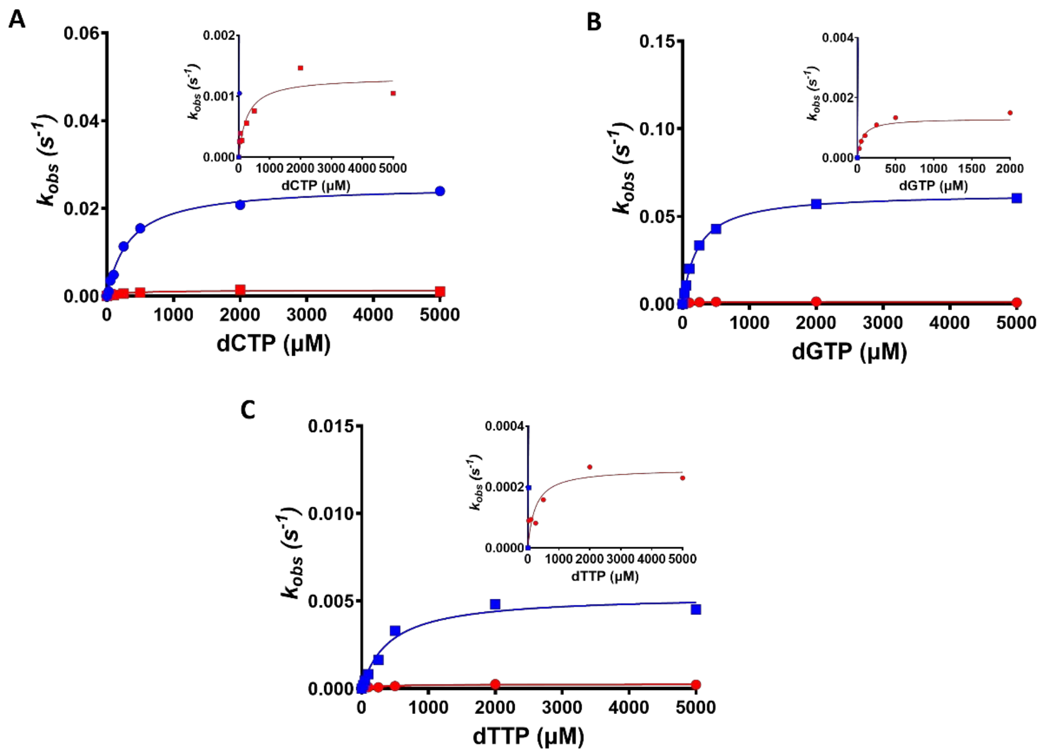

Supplementary Figure 6: Incorrect single turnover for T-template for WT (blue) and S180R (red) demonstrate that S180R is a slow variant under all sequence contexts, and with a similar dNTP apparent dissociation constant ($K_{d(dNTP)}$). dCTP (A), dGTP (B), and dTTP (C) data shown. Product formation over time was used to calculate a k_{obs} rate for each dNTP concentration (see representative panel in Supplementary figure 4). K_{obs} rates were then used to estimate the k_{pol} rates which are reported as mean value ± standard error in Table 1. The apparent dissociation constant for dNTP ($K_{d(dNTP)}$) was then estimated from the data and reported as mean value ± standard error in Table *1*. Data shown are representative plots, with a minimum sample size of two biological replicates per condition.

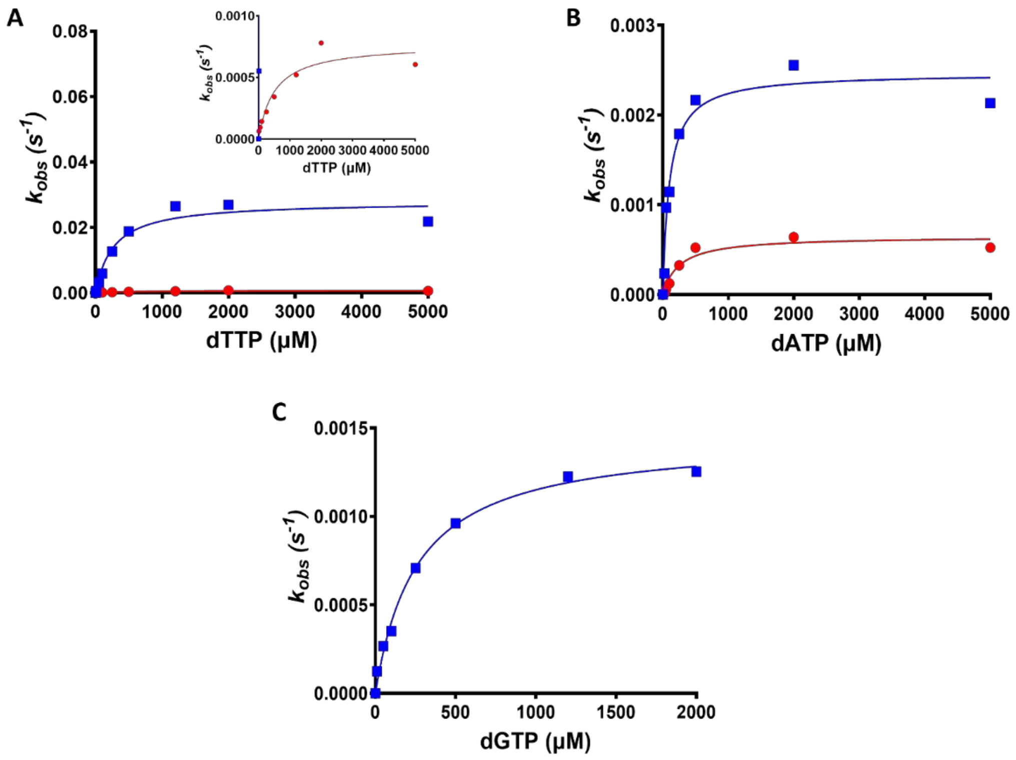

Supplementary Figure 7: Incorrect single turnover for G-template for WT (blue) and S180R (red) demonstrate that S180R is a slow variant under all sequence contexts, and with a similar dNTP apparent dissociation constant ($K_{d(dNTP)}$). dTTP (A), dATP (B), and dGTP (C) data shown, with exception that S180R does not insert dGTP opposite G-template (absence of red plot in C). Product formation over time was used to calculate a k_{obs} rate for each dNTP concentration (see representative panel in Supplementary figure 4). K_{obs} rates were then used to estimate the k_{pol} rates which are reported as mean value ± standard error in Table 1. The apparent dissociation constant for dNTP ($K_{d(dNTP)}$) was then estimated from the data and reported as mean value ± standard error in Table *1*. Data shown are representative plots, with a minimum sample size of two biological replicates per condition.

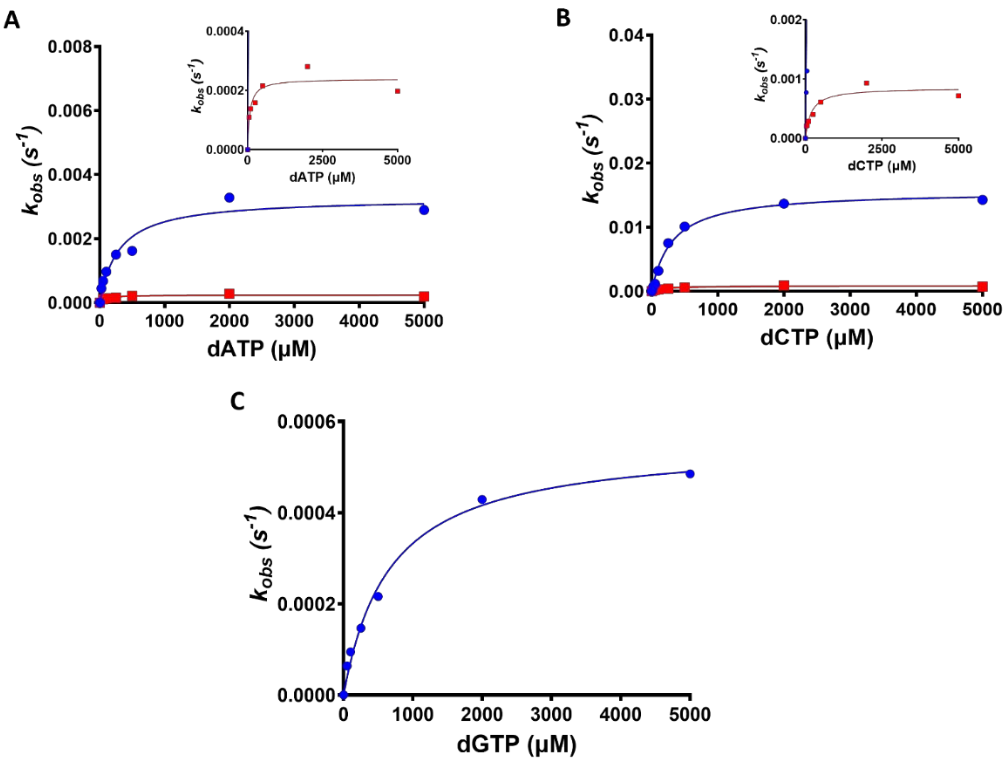

Supplementary Figure 8: Incorrect single turnover for A-template for WT (blue) and S180R (red) demonstrate that S180R is a slow variant under all sequence contexts, and with a similar dNTP apparent dissociation constant ($K_{d(dNTP)}$). dATP (A), dCTP (B), and dGTP (C) data shown, with exception that S180R does not insert dGTP opposite A-template (absence of red plot in C). Product formation over time was used to calculate a k_{obs} rate for each dNTP concentration (see representative panel in Supplementary figure 4). K_{obs} rates were then used to estimate the k_{pol} rates which are reported as mean value ± standard error in Table 1. The apparent dissociation constant for dNTP ($K_{d(dNTP)}$) was then estimated from the data and reported as mean value ± standard error in Table *1*. Data shown are representative plots, with a minimum sample size of two biological replicates per condition.

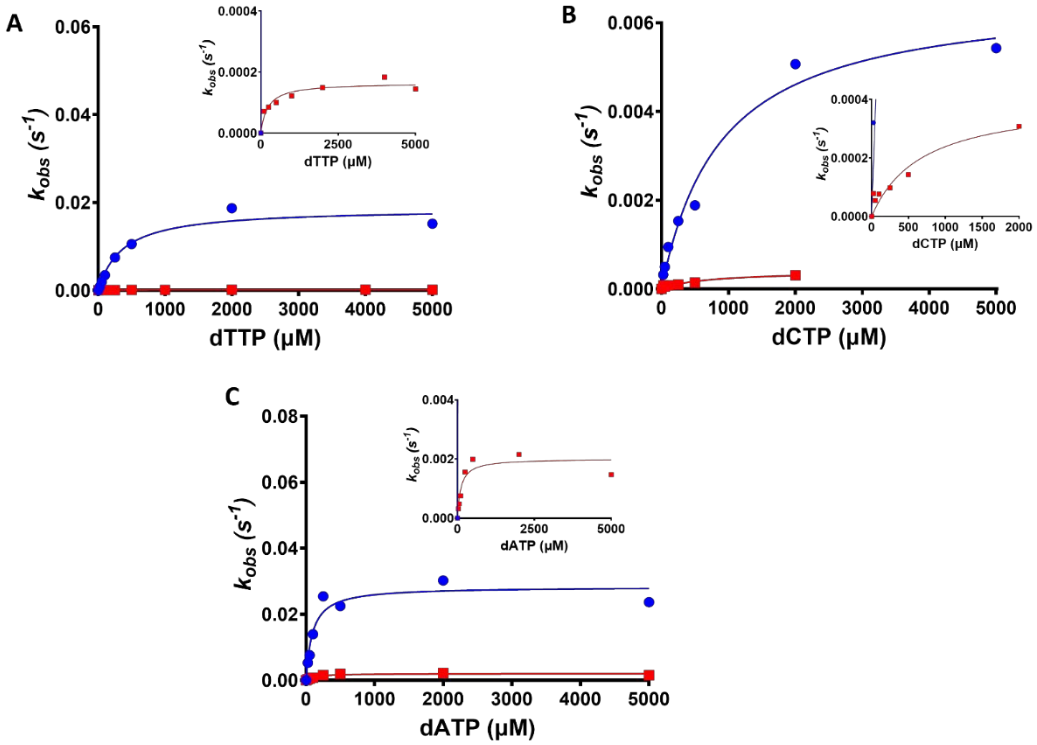

Supplementary Figure 9: Incorrect single turnover for C-template for WT (blue) and S180R (red) demonstrate that S180R is a slow variant under all sequence contexts, and with a similar dNTP apparent dissociation constant ($K_{d(dNTP)}$). dTTP (A), dCTP (B), and dATP (C) data shown. Product formation over time was used to calculate a k_{obs} rate for each dNTP concentration (see representative panel in Supplementary figure 4). K_{obs} rates were then used to estimate the k_{pol} rates which are reported as mean value ± standard error in Table 1. The apparent dissociation constant for dNTP ($K_{d(dNTP)}$) was then estimated from the data and reported as mean value ± standard error in Table *1*. Data shown are representative plots, with a minimum sample size of two biological replicates per condition.
